# Improving microRNA target prediction with gene expression profiles

**DOI:** 10.1186/s12864-016-2695-1

**Published:** 2016-05-17

**Authors:** Cesaré Ovando-Vázquez, Daniel Lepe-Soltero, Cei Abreu-Goodger

**Affiliations:** Unidad de Genómica Avanzada (Langebio), Centro de Investigación y de Estudios Avanzados del IPN, Irapuato, Guanajuato 36821 México

**Keywords:** microRNA target prediction, Support Vector Machine, Gene expression profiles, Biological context, microRNA perturbation experiments

## Abstract

**Background:**

Mammalian genomes encode for thousands of microRNAs, which can potentially regulate the majority of protein-coding genes. They have been implicated in development and disease, leading to great interest in understanding their function, with computational methods being widely used to predict their targets. Most computational methods rely on sequence features, thermodynamics, and conservation filters; essentially scanning the whole transcriptome to predict one set of targets for each microRNA. This has the limitation of not considering that the same microRNA could have different sets of targets, and thus different functions, when expressed in different types of cells.

**Results:**

To address this problem, we combine popular target prediction methods with expression profiles, via machine learning, to produce a new predictor: TargetExpress. Using independent data from microarrays and high-throughput sequencing, we show that TargetExpress outperforms existing methods, and that our predictions are enriched in functions that are coherent with the added expression profile and literature reports.

**Conclusions:**

Our method should be particularly useful for anyone studying the functions and targets of miRNAs in specific tissues or cells. TargetExpress is available at: http://targetexpress.ceiabreulab.org/.

**Electronic supplementary material:**

The online version of this article (doi:10.1186/s12864-016-2695-1) contains supplementary material, which is available to authorized users.

## Background

MicroRNAs (miRNAs) are small non-coding RNAs that guide Argonaute proteins to post-transcriptionally repress target messenger RNAs (mRNAs) [1]. In animals they act by binding mainly to sites in the 3’ untranslated region (3’UTR) of their targets that are complementary to the 5’ end of the miRNA (often called the “seed” region), causing translation repression and transcript destabilization [2]. There are 1881 annotated miRNA genes in the human genome [3], and these are predicted to target the majority of protein coding genes [4]. The large number of potential targets renders experimental validation a complicated task, and computational methods have risen as a useful alternative for high-throughput prediction.

Most computational methods to predict miRNA targets require the presence of 7-nucleotides of perfect complementarity to the seed region. Given the small size of these sites, many can occur just by chance, leading to false positive predictions. To increase the specificity of target prediction, computational methods have used other criteria, such as considering the presence of multiple binding sites or filtering by evolutionary conservation [5–7]. Additional sequence features have also been used to successfully prioritize functional sites, including their relative position within the 3’UTR, local structure or AU content and thermodynamic binding stability [4, 8–10]. More recently, a biophysical model was developed using the properties of experimentally determined Argonaute-bound sites [11]. An advantage of this model is that it can be used, in combination with conservation features, to accurately predict targets lacking the canonical perfect seed match [12]. All of these approaches are designed to predict targets across all known transcripts, without considering where miRNAs or potential targets are expressed.

There has recently been great interest in incorporating biological context to miRNA function prediction. Novel methods have been developed to take advantage of paired mRNA and miRNA expression data to infer the most relevant targets, based on the notion that the abundance of miRNAs should be inversely correlated with the expression of their targets [13–16]. While these methods can provide insights to the general function of many miRNAs across a range of biological contexts, they are only applicable when a relatively large amount of consistent experimental data is available, such as for the NCI-60 cancer cell line panel. Other methods use large sets of mRNA expression data across different conditions, without a corresponding miRNA profile, to select predicted targets that are consistently co-regulated [17, 18]. Although the above methods have been shown to provide target lists with more coherent biological annotations, they will necessarily miss targets that are only functional in a subset of the samples. More explicitly, none of these methods considers that the same miRNA could have different functions when acting within particular cell-types, where different transcripts are expressed at different concentrations, thus altering the potential target space.

Of particular relevance in this regard is TargetScore, which was designed to predict targets using miRNA-overexpression transcriptomic experiments [19]. These experiments are quite popular for determining the function of miRNAs in an unbiased way, and consistent methods for their analysis have not always been used [20–26]. The main drawback is that a new experiment needs to be performed every time a new combination of miRNA:cell-type would like to be evaluated. What would really be useful is a method that could take advantage of the over one million mRNA samples already profiled and available through the Gene Expression Omnibus [27]. The method should be able to take the gene expression profile for any cell-type, and predict the effect of overexpressing any miRNA, without having to perform the actual experiment.

Why would a miRNA have different functions in different cell-types? In the simplest case, a miRNA cannot repress a transcript that is not present in the same cell. Excluding predicted transcripts that are not expressed in the cell-type of interest can in principle solve this problem, although in practice this can be complicated to achieve. With microarrays, high background signal and non-specific probe hybridization can confound the detection of transcripts expressed at low levels. The digital nature of RNA-Seq alleviates these problems, but the absence of sequence from a particular transcript does not guarantee that the transcript is not present: sequencing to higher depth in general allows more transcripts to be detected [28, 29]. Let us assume that we can ignore this issue, and correctly define which potential targets are present in a particular type of cell. The abundance of these targets could still vary by 4 orders of magnitude [29], so can a miRNA equally repress them?

To approach this question, we first analysed microarray experiments where individual microRNAs had been perturbed. We observed that the magnitude with which potential targets are affected is not independent of their expression levels, with more abundant targets generally being more significantly affected (Additional file 1: Figure S1). This is consistent with a previous work showing that PAR-CLIP detected targets that were only bound to Argonaute in one type of B-cells tended to have higher expression levels in that cell type. Interestingly, many of these targets were expressed, but not bound to Argonaute, in another type of B-cells [30]. To take advantage of this relationship between expression level and miRNA target functionality, we decided to combine three “state of the art” miRNA target prediction methods based exclusively on sequence information (*TargetScan* [9], *microT-CDS* [10] and *MIRZA* [11]) with expression profiles from any cellular condition, using an SVM framework. We evaluated our method, *TargetExpress*, using independent microarray and RNA-Seq experiments. We show that our method performs better than individual target prediction methods at predicting the outcome of miRNA-perturbation experiments, and give examples of how our predictions can better reflect the biological function of a miRNA.

## Methods

### Microarray data

#### microRNA perturbation datasets

We downloaded raw data for miRNA over-expression (OE) or knock-down (KD) experiments in different cellular conditions from the Gene Expression Omnibus (GEO). Human experiments: hsa-miR-29 KD in IMR-90 foetal lung fibroblast cell line (GSE18651), hsa-miR-145 OE in MDA-MB-231 cells (GSE19737), hsa-miR-7 OE in A549 cells (GSE14507), hsa-miR-30 OE in 4L and 5B1 melanoma cell lines (GSE27718), hsa-miR-20a KD in non-tumorigenic epithelial MCF10A cell line (GSE33538), hsa-miR-122 KD in HES2 cell line (GSE13460), hsa-miR-34a OE in HCT116 cell line (GSE7754), hsa-miR-221 OE in PC-3 cell line (GSE45627), hsa-miR-483-5p OE in MMH-ES-1 cell line (GSE50980). Mouse experiments: mmu-miR-140 OE in C3H10T1/2 cell line (GSE13590), mmu-miR-122 KD in vivo liver cells (GSE13948), mmu-miR-290 cluster KD in ES cells (GSE8503), mmu-let-7 and mmu-miR-294 KD in ES cells (GSE18840), mmu-miR-29 OE in Astrocytes (GSE27035). Zebrafish experiments: dre-miR-1 and dre-miR-124 KD in GFP muscle and CNS cells (GSE12991), dre-miR-430 KD in embryo cells (GSE4201). We also obtained wild-type and mmu-miR-22 knockout (KO) mouse tissues from EBI ArrayExpress (E-MTAB-2038). Human and mouse experiments were used for training and during cross-validation. Zebrafish experiments were used to independently evaluate the prediction model.

We performed all data processing in R [31]. We normalized CEL files with RMA [32] using the *justRMA* function from the *affy* package [33]. To analyse differential expression we used *limma* [34]. For each microarray experiment, we defined the required contrast as “treatment” versus control. To facilitate comparisons we always considered the “treatment” sample to be the one with higher miRNA concentration, thus any OE was always the treatment but a KD became the control and the baseline (before KD) became the treatment. Additional file 1: Table S1 includes the exact contrasts that we used, and the number of differentially expressed genes detected with a 10 % False-Discovery Rate (FDR).

For each dataset we then verified that it captures a direct effect due to miRNA regulation. For this purpose we used Sylamer [21], that quantifies the over or under representation of miRNA seed matches in the 3’ UTRs of genes ordered by differential expression. The results for the evaluation datasets are included in Additional file 1: Table S1. Sylamer plots are shown in Additional file 1: Figure S2. We excluded the experiments that did not have a good Sylamer signal for the expected miRNA from all further analyses (Additional file 1: Table S1 and Figure S2). We believe this is an important filter that is not always undertaken, which allows us to focus on experimental results that truly reflect the direct targets of a miRNA.

### Independent control expression profiles

We also downloaded independent expression profiles (when available) from experiments that were not in the miRNA-perturbation datasets (see above) but represent the same cellular conditions. If there are no available independent expression profiles, we use control expression profiles from the experiment (Additional file 1: Table S2). We normalized these with RMA as above.

### MicroRNA target prediction data

We downloaded TargetScan v6.2 [9] predictions for human, mouse and zebrafish, selecting Total Context+ Score and Probability of Conserved Targeting (PCT) as prediction scores. Similarly, we downloaded microT-CDS [10] predictions for human and mouse, using miTG as the prediction score but only keeping predictions with positive UTR3 score (those with at least one target in the 3’UTR). For MIRZA predictions, we first downloaded human and mouse 3’UTR sequences from http://www.targetscan.org/vert_70/vert_70_data_download/UTR_Sequences.txt.zip and http://www.targetscan.org/mmu_61/mmu_61_data_download/UTR_Sequences.txt.zip respectively. Following recommendations from the MIRZA publication, we split each 3’UTR into 50 nt windows, shifting 25 nt at each step [11]. We obtained alternative MIRZA scores (canonical sites - MIRZA, target frequency - MIRZA-F and non canonical sites - MIRZA-N) for each transcript by summing the logarithms of the target scores of all sites in the transcript [11].

### SVM features and training: TargetExpress

To train the model we first defined which predicted targets are True Targets (TT) and which are False Targets (FT), based on the differential expression results of the miRNA-perturbation datasets. Only human and mouse datasets were used for this purpose, since both species have TargetScan, microT-CDS and MIRZA predictions. For each experiment we defined TT as those predicted targets that were repressed (log_2_ Fold-change < 0) significantly (FDR < 0.1) by the miRNA. We defined the FT as all the predicted targets with an FDR > 0.2 irrespective of the direction of change.

The features we used for each target consisted of target prediction scores – from TargetScan: Total Context+ Score and Probability of Conserved Targeting (PCT), from microT-CDS: miTG, from MIRZA: MIRZA canonical sites (MIRZA), MIRZA target frequency (MIRZA-F) and MIRZA non canonical sites (MIRZA-N) – and an expression value for a particular cellular condition. For each experiment we only analyse transcripts that have either a TargetScan, microT-CDS or MIRZA score. Individual transcripts may not have one of the Total Context+, miTG, PCT, MIRZA, MIRZA-F or MIRZA-N scores. Instead of discarding these transcripts, we assign a neutral value to the missing scores (see below), enabling them to be used during training.

We only consider transcripts with a label (TT or FT), ignoring predicted targets that were not measured in the experiment. This yields a processed matrix for each miRNA-perturbation experiment, with the following training features: Total Context + Score, PCT, miTG, MIRZA, MIRZA-F, MIRZA-N and expression value. We rank each feature of this training matrix and scale them using the min-max normalization. Since we scale using a min-max normalization between −1 and +1 we substitute missing scores with the normalization min-max centre, in this case 0.

We use these feature-ranked matrices to train *n* SVM classification models [35] using the *e1071 R package* [36]. In the current implementation *n* corresponds to the 13 experiments with good Sylamer signal and at least 20 True Targets (Additional file 1: Table S1). We call the combination of these *n* SVM classification models “TargetExpress”. Feature ranking allows us to use different types of expression values (that can have different range and distributions) such as those coming from microarray, RNA-Seq or qPCR. We decided to use a radial kernel to create the *n* SVMs. In particular we use the function *svm* with *gamma* = 1/7, cost = 1, with the radial kernel and scale set to FALSE since our data is already scaled (min-max normalized). In the prediction stage, we sum the predictions of each *n* SVM models given an evaluation dataset. This prediction sum (consensus) becomes the final TargetExpress prediction score.

Each training data set suffers from class imbalance, with many more FT than TT, so we performed a so-called “class weighting scheme” as implemented in the *e1071* R package [36]. Penalization parameters for each class are defined as ${C}_1=\frac{1}{n_1}*\frac{1}{sum\;\left(1/{n}_1\right)}$ and ${C}_2=\frac{1}{n_2}*\frac{1}{sum\;\left(1/{n}_2\right)}.$ Where ${n}_1$ is the number of TT and ${n}_2$ the number of FT.

### Evaluating predictions

We used the Area Under the Curve (AUC) of Receiver Operating Characteristic (ROC) curves to evaluate our predictions [37, 38]. Each AUC evaluation is performed by the *roc* function from the *pROC* R package [39]. AUC can be interpreted as the “probability that the classifier will rank a randomly chosen positive instance higher than a randomly chosen negative instance” [40]. Sensitivity and specificity for each ROC were calculated by first finding the minimum distance from the curve to the top left corner (sensitivity = 1, specificity = 1).

### TargetExpress validation and performance

To evaluate the robustness of our SVM classification model (TargetExpress) we varied the True Target definition “t < 0 and FDR < fdr” by selecting fdr = 0.01, 0.05, 0.1 and 0.2 to observe how the FDR choice can affect the SVM prediction performance. We decided to evaluate the performance of our model using both cross and independent validation. Cross-validation consists of leaving out data from one experiment, re-training with the other *n-1* experiments then testing against the left-out experiment. These results are presented in Additional file 1: Figure S3.

We selected various combinations of methods to test the performance of our model. The “intersect” approach, for any particular target prediction method, consists of only considering targets with higher expression than the median of the whole microarray. This is a quick way of removing genes that are not expressed in the particular experimental context. We also generated versions of TargetExpress that only consider one of the sequence-based target prediction methods, by setting the other scaled scores to the neutral value 0. In total, we calculated AUCs for each left-out experiment using the following methods or combinations: TargetScan, TargetScan-intersect and TargetExpress using only TargetScan scores; microT-CDS, microT-CDS-intersect and TargetExpress using only microT-CDS scores; a sum of TargetScan and microT-CDS scores, sum of TargetScan and microT-CDS scores-intersect and TargetExpress using both TargetScan and microT-CDS scores. Here we selected only TargetScan and microT-CDS scores given they have the top two AUC scores (Additional file 1: Figure S3).

Since we used the human and mouse data to train TargetExpress, we also wanted to evaluate its performance against a truly independent dataset. We selected zebrafish experiments performed under three different cellular conditions: developing embryo, and cell-sorted central nervous system or muscle cells. These experiments were designed to discover targets regulated by dre-miR-430, dre-miR124 and dre-miR-1/133, respectively. We defined TT and FT as before.

### Target predictions for present genes

A simplistic approach to obtain cell-type specific targets, mentioned in the Introduction, is working only with genes that are detected as being expressed (or “present”) in the condition of interest. To test this idea in a more sophisticated manner than the “intersect” approach mentioned above, we restricted target predictions for zebrafish to those called “present” in each condition using the *mas5calls* function from the *affy* R package [33].

### Validation with RNA-Seq data

To test our method with a different kind of expression data, we downloaded the raw sequences from a Zebrafish RNA-Seq and ribosome profiling experiment designed to discover dre-miR-430 targets (Short Read Archive SRP010040/GEO accession GSE34743) [2]. We converted the Short Read Archive files (SRA) to fastq format using *fastq-dump* from the SRA toolkit [41]. All samples were 3’-adapter trimmed using *reaper* [42], then mapped to the zebrafish genome (Zv9) downloaded from Ensembl [43] using Bowtie 2 [44]. We quantified gene expression in all samples by overlapping the mapped reads to gene locations, using the *GenomicFeatures* R package [45]. We analysed both types of experiment: mRNA-Seq and ribosome protected fragments (ribosome profiling). For both kinds of profiles we performed differential expression analysis using the *edgeR* R package [46]. The results for the differential expression analysis are included in Additional file 1: Table S1. We then defined True Targets and False Targets, and calculated AUCs, as for the microarray experiments.

### Comparing predictions with microarray or RNA-Seq expression data

To compare which technology is better for our method, we would ideally want RNA from the same experiment analysed by both microarray and RNA-Seq. Since this is not available, we used two different experiments that studied mouse B-cells in wild-type and miR-155 knockout conditions. The microarray experiment is available through ArrayExpress (E-MEXP-1325) and consists of purified mouse B cells (wild-type and miR-155-KO) stimulated with LPS and IL4 for 24 h (to induce miR-155 expression) [47]. We processed the CEL files as described above for all other microarray experiments. The RNA-Seq experiment is available through GEO (GSE61425) and consists of purified mouse B cells (wild-type and miR-155-KO) stimulated with LPS and IL4 for 4 days [48]. To facilitate the comparison of this RNA-Seq experiment to the microarray one, we processed the SRA files as described above, with the following modifications. We used Kallisto to perform mapping and quantification of the fastq files simultaneously [49]. We then used *voom* to transform the estimated counts to values that are amenable to processing with standard microarray linear models [50]. After this, we performed differential expression analysis with *limma* in the same manner as for the other microarray experiments. To make comparisons more even during ROC analysis, we defined the True Targets for both experiments as the top 250 down-regulated genes (t-statistic < 0) and the top 250 non-changing genes (abs(t) < 1) as False Targets.

### Gene Set functional enrichment tests for TargetExpress predictions

We downloaded normal tissue mRNA-Seq data from the Human Body Map 2.0 [51] consisting of single and paired-end reads. Single-end sets consist of 65–84 million 75 nt reads per sample. Paired-end sets consist of 64–84 million 50 nt read pairs per sample. All samples were mapped to the human genome (Ensembl hg19) [43] using Bowtie 2 [44]. Once mapped to hg19, we obtained counts for each gene per sample by overlapping the mapped reads to gene locations in the genome using the *GenomicFeatures* R package [45]. The raw counts in each tissue and gene lengths are used to calculate Reads Per-Kilobase of gene per Million mapped (RPKM) values. The RPKMs are used as the expression values of each gene in each tissue.

We applied our model to predict targets for hsa-miR-29 in two different tissues: heart and brain. We selected this miRNA because it is highly expressed in several adult tissues; we focus on these two particular tissues, since they have very different gene expression profiles. As a negative control we randomized the original TargetExpress heart and brain prediction scores.

To perform Gene Set enrichments tests on our predicted targets in different cellular conditions, we used the *wilcoxGST* test from the *limma* R package (described in [52]). We defined the universe for the Gene Set Test as the union of targets predicted by Target Scan, microT-CDS (miTG 3UTR score > 0), MIRZA, MIRZA-F and MIRZA-N. To simplify interpretation, we only used Biological Process GO Terms with more than 5 and less than 100 genes.

### TargetExpress web interface

In addition to the standalone version of our model, we provide a simple web interface so that users can explore TargetExpress predictions. The user needs to provide three things: i) the miRNA of interest, ii) the target prediction method (TargetScan, microT-CDS and/or MIRZA predictions), and iii) an expression profile with RefSeq transcript ids. We provide a link so that users can use DAVID [53, 54] to transform different kinds of gene identifiers into the required RefSeq ids. Currently, the web interface includes TargetScan, microT-CDS and MIRZA predictions for human and mouse.

## Results

### Training a Support Vector Machine

**Fig. 1:**
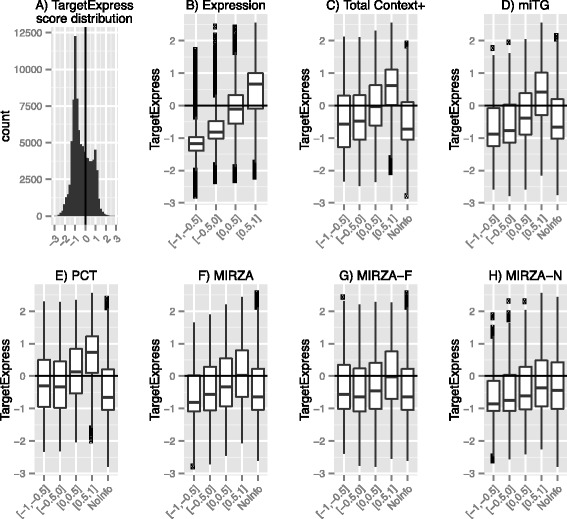
Contribution of features to SVM performance. **a** TargetExpress score prediction distribution, with positive numbers representing predicted True targets for a particular expression context. Features consist of: **b** Expression, **c** TargetScan Total Context+, **d** microT-CDS miTG, **e** TargetScan Probability of Conserved Targeting (PCT), **f** MIRZA, **g** MIRZA-F and **h** MIRZA-N. Scaled score ranges for each feature ([−1. -0.5], [−0.5, 0], [0, 0.5], [0.5, 1]) used to build the SVM models contribute differently to predict True targets. When there are transcripts with no information for a particular feature (C-H), we assign them the scaled min-max centre, in this case 0

We developed a Machine Learning classification model (TargetExpress) to improve available miRNA target predictions by including expression profiles (see Methods). TargetExpress gives positive and negative scores. Positive scores indicate predicted True miRNA targets and negative scores indicate False targets (Fig. 1a).

After training our *n* SVM models (see Methods), we can interpret how each feature contributes to the final prediction (TargetExpress score), by observing the Area Under the Curve (AUC) for each feature within each SVM model (Additional file 1: Figure S3). The expression feature has the highest weight (Fig. 1b and Additional file 1: Figure S3), telling us that expression substantially helps to predict True targets. This is expected since non-expressed targets (not present) cannot be repressed. Since there is no perfect way to define expressed and non-expressed genes in a microarray experiment (due to background noise and cross-hybridization), our SVM approach is a useful alternative. In addition, mRNAs with low expression levels are less likely to be experimentally detected as True miRNA targets. This is visible in Fig. 1b, where targets with normalized expression values between 0.5-1 have higher TargetExpress scores than those between 0–0.5, even though both likely consist of expressed genes. This could be a statistical artefact, due to the fact that low expression is inherently noisier and thus there is less power to detect a significant change. In any case, TargetExpress was designed to predict the outcome of the experiment, and thus takes advantage of the full range of expression values to provide improved target predictions.

In Fig. 1 we can also observe that features do not contribute equally to predict True miRNA targets. From Fig. 1c-h we can see that TargetScan, microT and MIRZA predictions in the high-score range (0.5-1) contribute to the functional TargetExpress predictions (positive scores), yet still include many targets with negative scores (Additional file 1: Table S3). In comparison, very few lowly expressed targets are predicted to be functional. This suggests that imposing any fixed score threshold for TargetScan, microT or MIRZA will not drastically improve predictions, but imposing a fixed threshold using expression data should.

### Cross-validation of TargetExpress

**Fig. 2:**
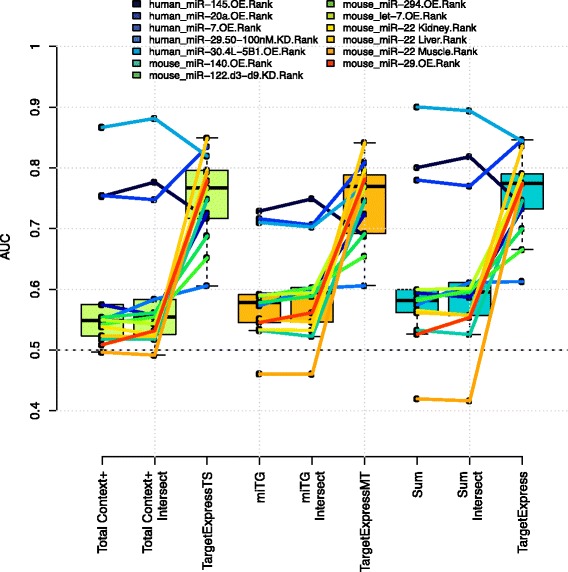
Leave one out cross-validation. The area under the curve (AUC) for each “left out” experiment (indicated in the top legend) given different prediction models: TargetScan, TargetScan-intersect and TargetExpress-TS (*green boxplots*); microT, microT-intersect and TargetExpress-MT (*orange*); Sum, Sum-intersect and TargetExpress-Sum (*blue*)

SVM models can easily be over-fit to the training data, so we first evaluated our model using a “leave one out” cross-validation. This consists of removing one experiment before training with the rest, and evaluating the performance of the new *n-1* SVM models against the left-out experiment. For this evaluation we built *n-1* SVM models for the individual TargetScan (TS) and microT (MT) predictions, as well as their “Sum” (see Methods), on their own or in combination with mRNA expression values (TargetExpress predictions). Here we decided to focus on TS and MT predictions since they showed the best performance. For each of the three initial methods (TS, MT and Sum) we also applied a simple “intersect” approach (considering only targets with expression greater than the median, see Methods). This led to a total of 9 different models that we compared in groups of three (Fig. 2, Additional file 1: Figure S4).

We found that TargetExpress-TS significantly outperformed both TS and TS-intersect, *p*-value = 0.0006, and 0.0012 (one-sided Wilcoxon Rank Sum paired test). TargetExpress-MT outperforms MT and MT-intersect as well, *p*-value = 0.0004 and 0.0006). The combined TargetExpress-Sum also performed significantly better than Sum and Sum-intersect (*p*-value = 0.0012 and 0.0023). Importantly, TargetExpress performs slightly better than TargetExpress-TS or TargetExpress-MT, (*p*-value = 0.0955 and 0.0549). This result suggests that combining different target prediction methods can improve microRNA target predictions. However simply adding expression information leads to a large improvement over any individual target predictor.

In Additional file 1: Figure S5 we show that TargetExpress models have higher AUCs than individual TargetScan or microT-CDS predictions, and also a better balance between sensitivity and specificity.

### Independent validation and specificity for different cellular conditions

The best way to evaluate an SVM is with fully independent data. For this, we decided to use zebrafish microarray experiments: a rigorous performance test, since we only used human and mouse data to train TargetExpress. There are no microT-CDS predictions available for zebrafish, so we tested if our model improves TargetScan predictions, and makes them more specific to particular cellular conditions.

We selected the following zebrafish experiments with a strong Sylamer signal (see Methods and Additional file 1: Table S1) to evaluate the specificity of our SVM model: knock-down of miR-1 and miR-124 in muscle and central nervous system cells respectively (GSE12991), and knock-down of miR-430 in embryo cells (GSE4201). With the control condition expression profiles from the same experiments (muscle, CNS, embryo) we generated predictions for each miRNA (miR-1, miR-124, miR-430), for a total of 9 sets of targets. We generated all possible combinations of miRNAs and tissues to use the “incorrect” tissues as negative controls. Then, we evaluated these predictions given the True Targets (TT) and False Targets (FT) defined from the differential expression analysis of the knock-down experiments (see Methods).

Our predictions with the correct cellular condition improve on the original TargetScan scores and also perform better than when any of the incorrect expression profiles are used (Additional file 1: Figure S6). From this, we conclude that our model generally improves TargetScan predictions making them more specific to a selected cellular condition.

**Fig. 3:**
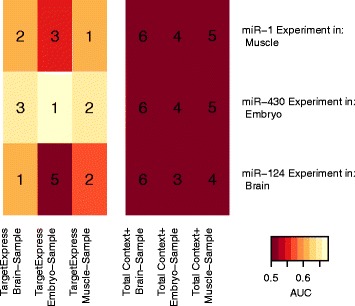
Tissue specificity performance of predictions in zebrafish, restricted to significantly expressed genes. For each miRNA (rows), we compared six prediction sets (columns): TargetExpress predictions for three different tissues, and TargetScan predictions for transcripts present in each tissue. Each result is evaluated using the AUC metric and given a rank according to it’s relative position amongst the six methods that we compared (1 = best, 6 = worst)

A common practice to improve predictions is to restrict them to the expressed transcripts, since if a transcript is not present it clearly cannot be targeted. Although defining expressed or “present” transcripts using microarray results is error prone, for this example we used an approach defined by Affymetrix: probesets that have significantly higher signal than their matched negative controls [55] (see Methods and previous section). We thus compare our predictions against TargetScan, but restricting the whole analysis to transcripts that are deemed to be “present” in each experiment, using the same zebrafish dataset. TargetExpress still outperforms the original TargetScan scores in all three cellular conditions, suggesting that it’s improvement is not simply due to ignoring non-expressed targets (Fig. 3).

### Applying TargetExpress to RNA-Seq data

During training TargetExpress used only microarray data, so we wanted to demonstrate that it works well on other types of expression profiles. We selected another zebrafish experiment, this time using high-throughput sequencing to measure expression [2]. The experiment consists of comparing wild-type zebrafish embryos to maternal zygotic Dicer mutants at several development time points. During this period a single microRNA, miR-430, dramatically increases expression under normal conditions. It is also an interesting experiment since it used RNA-Seq and Ribosome-profiling (a proxy to measure translation), to detect miRNA repression affecting either transcript levels or translation.

**Fig. 4:**
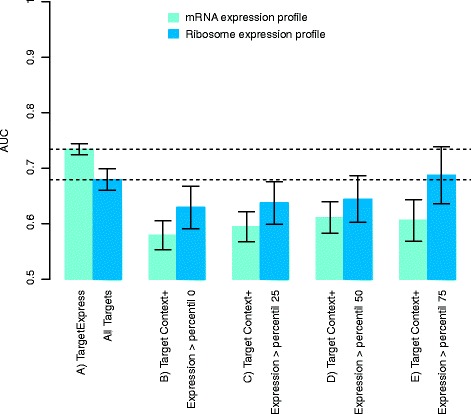
Performance comparison using high-throughput sequencing data of zebrafish embryos. Experimental profiles compared wild type and dicer mutant embryos at 6 h postfertilization, when a clear difference of miR-430 targets is observed [2]. We compared **a**) TargetExpress predictions to TargetScan restricted to targets with expression higher than percentiles: **b**) 0, **c**) 25, **d**) 50 and **e**) 75. The Y-axis shows the Area Under the Curve metric (AUC), with 95 % confidence intervals. Green bars represent performance when using mRNA expression, blue bars ribosome profiling. Dashed lines indicate TargetExpress AUCs

We can see that TargetExpress again substantially improves the original TargetScan scores, when including mRNA or Ribosome profiling expression measurements (Fig. 4). This improvement can be partially explained since True targets in the control samples (MZDicer mutants, lacking miR-430 expression) tend to be highly expressed (Additional file 1: Figure S7). Nevertheless, even when analysing transcripts with increasing expression level (above percentiles 25, 50 or 75) TargetScan only slightly improved its performance (Fig. 4, Additional file 1: Figures 8 and 9). In all these cases, TargetExpress consistently improves on the single target prediction method.

### Comparing predictions using microarray or RNA-Seq expression profiles

**Fig. 5:**
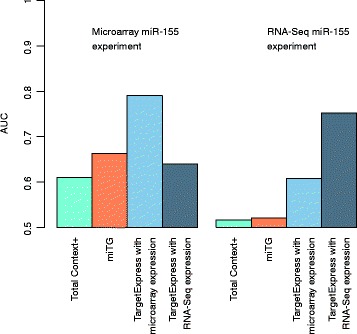
Performance comparison using microarray or RNA-Seq expression from similar experiments. Two different experiments used microarray or RNA-Seq to detect expression changes in mouse miR-155 knockout B cells [47, 48]. We compared TargetScan and microT predictions to TargetExpress using either microarray or RNA-Seq expression profiles. The Y-axis shows the Area Under the Curve metric (AUC)

We next wanted to test the effect of using microarray or RNA-Seq expression profiles with TargetExpress. Unfortunately, we do not have a single experiment where the same RNA samples were profiled using both technologies. The closest we found were two experiments comparing mouse wild-type and miR-155 knockout B cells performed under similar conditions (see Methods). Both experiments had a good number of biological replicates, and the direct effect of miR-155 was clearly detected using Sylamer analysis (Additional file 1: Figure S10). The Sylamer profile for the microarray experiment shows a very steep enrichment peak (Additional file 1: Figure S10A), suggesting that direct targets are more easily detected than in the RNA-Seq experiment where a broader peak implicates a combination of direct targets with secondary effects (Additional file 1: Figure S10B). This is consistent with the RNA-Seq experiment being performed 4 days after miR-155 activation, as opposed to 1 day for the microarray experiment. Due to these differences, we decided against trying to compare the actual genes detected as targets in each experiment. Nevertheless, what is clear is that TargetExpress can successfully use either kind of expression profile, achieving better predictions than stand-alone target prediction methods (Fig. 5). Also, the performance is better when using the control profile from the actual experiment (microarray or RNA-Seq).

### Different miRNA functions in different tissues

In the previous sections, we showed that TargetExpress improves prediction of microRNA targets, achieving its best performance when using the appropriate expression profile. We were curious to see if TargetExpress allows us to describe different biological functions for the same microRNA, when expressed in different tissues. We focused on miR-29, a highly conserved microRNA that is expressed in many tissues (http://mellfire.ugent.be/public/body_map/). We obtained expression profiles for 16 different tissues from the Human Body Map 2.0 [51] (see Methods). As an example, we chose two tissues that express miR-29 but with very different mRNA expression profiles: brain and heart. The idea is to highlight what makes our model unique: predicting different functions, within different tissues, for a single miRNA.

**Fig. 6:**
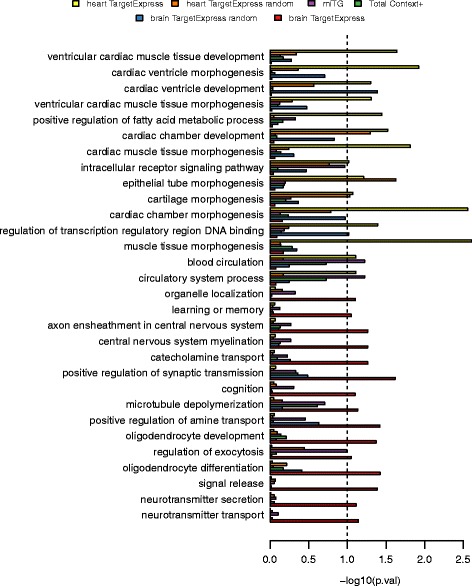
GO term enrichment comparing predictions for miR-29 in heart and brain tissues. GO enrichment for TargetExpress predictions are shown in yellow (heart) and red (brain) bars. Random TargetExpress predictions are shown in orange (heart) and blue (brain). GO enrichment values for microT-CDS and TargetScan predictions are shown in purple and green bars, respectively. Full GO enrichment results are in Additional file 2: Table S4

For a systematic test, we performed GO term enrichment analysis (Additional file 1: Table S4), and then selected terms that were differentially enriched between heart and brain target predictions. As expected, several of the most significant GO terms are related to the underlying tissue. The TargetExpress miR-29 brain predictions were enriched for terms such as “neurotransmitter transport/secretion”, “oligodendrocyte differentiation/development”, “oligodendrocyte development”, “cognition” and “learning or memory. These terms were not as enriched for the other prediction methods (Total Context+, miTG or a random brain TargetExpress control). On the other hand, the TargetExpress miR-29 heart predictions included terms like “cardiac ventricle morphogenesis/development”, “cardiac chamber development” and “cardiac muscle tissue development” that were also not so enriched for the other methods (Fig. 6). We also searched the literature for articles studying miR-29 function. Previous evidence suggests that miR-29 down-regulates heart functions related to fibril and collagen [56, 57], and brain functions related to cellular death and apoptosis [58, 59]. These also appear in our enriched GO terms, where we find functions related to collagen and myofibril assembly in TargetExpress heart predictions, and functions related to neuron apoptosis in TargetExpress brain predictions (Additional file 1: Figure S11).

## Discussion

There is great interest to incorporate more biological context to microRNA target predictions. Unfortunately, many methods that do so require a large number of expression profiles [17, 18] or series of matched mRNA and miRNA profiles [13–16]. We developed TargetExpress to improve available predicted targets by adding a single mRNA expression profile, making the targets more specific to particular cellular conditions. Our method consists of ranking and scaling target predictions and expression values, then combining (adding up) the predictions of *n* SVM classification models (Additional file 1: Table S1). The new predictions can be seen as a non-linear filter for each original prediction and expression value. Results of this “filtering” are those mRNAs that are predicted to be more down regulated by a miRNA in a specific cellular condition.

Regular target predictions only use sequence features, making them very sensitive but unable to differentiate targets that are repressed mainly in specific cellular conditions. In order to obtain miRNA targets relevant to a particular cell-type, a simple and commonly used strategy is to select only the transcripts that are robustly expressed (typically transcripts with expression greater than the median). We compare TargetExpress against three of the most popular target prediction methods. Even when only analysing expressed transcripts, TargetExpress performs better than the original target predictions. However, during cross-validation we observed that two experiments did not benefit from adding expression data: the overexpression of miR-30a and miR-145 in human cancer cell lines (Fig. 2). Interestingly, the raw methods already have extremely high performance for these cases, suggesting a limit above which adding expression context will no longer improve predictions. Nevertheless, when we tested TargetExpress using completely independent datasets (zebrafish microarrays and RNA-seq/ribosome profiling) we observed better performance than TargetScan.

TargetExpress helps us refine available target predictions, selecting those transcripts that are more likely to be repressed in specific cellular conditions, by including gene expression measurements. One of the most interesting applications of our method is to generate different lists of targets for the same miRNA, using expression profiles from different tissues or cells. As an example of this, we present results for miR-29 predictions for heart and brain tissues, showing that enriched GO terms are more consistent with these tissues and with published results.

## Conclusions

Our method is intended for anyone interested in the function of miRNAs in specific cell-types. The ideal experiment in these cases is to overexpress or knockdown the miRNA of interest in the cell-type of interest, followed by genome-wide expression profiling [22, 24, 26]. Lists of targets can then be derived as we described (see Methods) or by using TargetScore, a method that combines fold-change measurements and sequenced-based scores [19]. When such experiments are not available, or cannot be performed for cost or time reasons, TargetExpress is a useful alternative, since it only needs predicted target scores for a miRNA and expression measurements for these potential targets in the condition of interest. It can thus be used to systematically predict the outcome of overexpression or knockdown experiments across a large number of tissues or cell lines.

In addition to a stand-alone program, we provide a web site that includes TargetScan, microT-CDS and MIRZA predictions for human and mouse miRNAs. The user only needs to select their favourite miRNA and upload an expression profile. Additional links allow annotation and functional enrichment analyses, using the DAVID website [53]. TargetExpress is available at: http://targetexpress.ceiabreulab.org/.

### Ethics

Not applicable.

### Consent to publish

Not applicable.

### Availability of data and materials

All the data used in this study was obtained from public databases, as described in Additional file 1: Supplementary Material.

## Additional files

Additional file 1: Supplementary Material. This file contains **Tables S1–S3** and **Figures S1-S11**. These include full descriptions and QC of the microarray and RNA-Seq experiments that we analysed, the number of training genes contributing to the TargetExpress model, expression bias of functional miRNA targets, Sylamer seed-enrichment results, additional performance comparisons between target prediction models (AUC, sensitivity, specificity and ROC curves) and enriched GO Terms suggested by miR-29 literature. (PDF 1937 kb)

Additional file 2: Table S4. Gene Ontology enrichment results for miR-29 TargetExpress predictions using brain or heart expression profiles. (XLSX 346 kb)

## Abbreviations

3’UTR: 3’ untranslated region
AUC: area under the curve
FDR: False-Discovery Rate
FT: false targets
GEO: Gene Expression Omnibus
KD: knock-down
KO: knockout
MIRZA-F: MIRZA target frequency
MIRZA-N: MIRZA non canonical sites
MT: microT
OE: over-expression
PCR: probability of conserved targeting
ROC: receiver operating characteristic
RPKM: reads per-kilobase of gene per million mapped
SRA: short read archive files
TS: targetscan
TT: true targets
mRNA: messenger RNA
miRNA: micro RNA

## References

[1] Bartel DP (2009). MicroRNAs: target recognition and regulatory functions. Cell.

[2] Bazzini AA, Lee MT, Giraldez AJ (2012). Ribosome Profiling Shows That miR-430 Reduces Translation Before Causing mRNA Decay in Zebrafish. Science.

[3] Kozomara A, Griffiths-Jones S (2014). miRBase: Annotating high confidence microRNAs using deep sequencing data. Nucleic Acids Res.

[4] Friedman RC, Farh KK-H, Burge CB, Bartel DP (2009). Most mammalian mRNAs are conserved targets of microRNAs. Genome Res.

[5] Lewis BP, Burge CB, Bartel DP (2005). Conserved seed pairing, often flanked by adenosines, indicates that thousands of human genes are microRNA targets. Cell.

[6] Krek A, Grün D, Poy MN, Wolf R, Rosenberg L, Epstein EJ, MacMenamin P, da Piedade I, Gunsalus KC, Stoffel M, Rajewsky N (2005). Combinatorial microRNA target predictions. Nat Genet.

[7] Enright AJ, John B, Gaul U, Tuschl T, Sander C, Marks DS (2003). MicroRNA targets in Drosophila. Genome Biol.

[8] Grimson A, Farh KK-H, Johnston WK, Garrett-Engele P, Lim LP, Bartel DP (2007). MicroRNA targeting specificity in mammals: determinants beyond seed pairing. Mol Cell.

[9] Garcia DM, Baek D, Shin C, Bell GW, Grimson A, Bartel DP (2011). Weak seed-pairing stability and high target-site abundance decrease the proficiency of lsy-6 and other microRNAs. Nat Struct Mol Biol.

[10] Reczko M, Maragkakis M, Alexiou P, Grosse I, Hatzigeorgiou AG (2012). Functional microRNA targets in protein coding sequences. Bioinformatics.

[11] Khorshid M, Hausser J, Zavolan M, van Nimwegen E (2013). A biophysical miRNA-mRNA interaction model infers canonical and noncanonical targets. Nat Methods.

[12] Gumienny R, Zavolan M (2015). Accurate transcriptome-wide prediction of microRNA targets and small interfering RNA off-targets with MIRZA-G. Nucleic Acids Res.

[13] Huang JC, Babak T, Corson TW, Chua G, Khan S, Gallie BL, Hughes TR, Blencowe BJ, Frey BJ, Morris QD (2007). Using expression profiling data to identify human microRNA targets. Nat Methods.

[14] Wang Y-P, Li K-B (2009). Correlation of expression profiles between microRNAs and mRNA targets using NCI-60 data. BMC Genomics.

[15] Naifang S, Minping Q, Minghua D (2013). Integrative Approaches for microRNA Target Prediction: Combining Sequence Information and the Paired mRNA and miRNA Expression Profiles. Curr Bioinform.

[16] Bossel Ben-Moshe N, Avraham R, Kedmi M, Zeisel A, Yitzhaky A, Yarden Y, Domany E (2012). Context-specific microRNA analysis: identification of functional microRNAs and their mRNA targets. Nucleic Acids Res.

[17] Gennarino VA, D’Angelo G, Dharmalingam G, Fernandez S, Russolillo G, Sanges R, Mutarelli M, Belcastro V, Ballabio A, Verde P, Sardiello M, Banfi S (2012). Identification of microRNA-regulated gene networks by expression analysis of target genes. Genome Res.

[18] Radfar H, Wong W, Morris Q (2013). BayMiR: inferring evidence for endogenous miRNA-induced gene repression from mRNA expression profiles. BMC Genomics.

[19] Li Y, Goldenberg A, Wong K-C, Zhang Z (2014). A probabilistic approach to explore human miRNA targetome by integrating miRNA-overexpression data and sequence information. Bioinformatics.

[20] Lim L, Lau N, Garrett-Engele P, Grimson A, Schelter J, Castle J, Bartel D, Linsley P, Johnson J (2005). Microarray analysis shows that some microRNAs downregulate large numbers of target mRNAs. Nature.

[21] van Dongen S, Abreu-Goodger C, Enright AJ (2008). Detecting microRNA binding and siRNA off-target effects from expression data. Nat Methods.

[22] Melton C, Judson RL, Blelloch R (2010). Opposing microRNA families regulate self-renewal in mouse embryonic stem cells. Nature.

[23] Wang WX, Wilfred BR, Xie K, Jennings MH, Hu YH, Stromberg AJ, Nelson PT (2010). Individual microRNAs (miRNAs) display distinct mRNA targeting “rules.”. RNA Biol.

[24] Davis MP, Abreu-Goodger C, van Dongen S, Lu D, Tate PH, Bartonicek N, Kutter C, Liu P, Skarnes WC, Enright AJ, Dunham I (2012). Large-scale identification of microRNA targets in murine Dgcr8-deficient embryonic stem cell lines. PLoS One.

[25] Eichhorn SW, Guo H, McGeary SE, Rodriguez-Mias RA, Shin C, Baek D, Hsu S, Ghoshal K, Villén J, Bartel DP (2014). mRNA Destabilization Is the Dominant Effect of Mammalian MicroRNAs by the Time Substantial Repression Ensues. Mol Cell.

[26] Santhakumar D, Forster T, Laqtom NN, Fragkoudis R, Dickinson P, Abreu-Goodger C, Manakov SA, Choudhury NR, Griffiths SJ, Vermeulen A, Enright AJ, Dutia B, Kohl A, Ghazal P, Buck AH (2010). Combined agonist–antagonist genome-wide functional screening identifies broadly active antiviral microRNAs. Proc Natl Acad Sci U S A.

[27] Barrett T, Wilhite SE, Ledoux P, Evangelista C, Kim IF, Tomashevsky M, Marshall KA, Phillippy KH, Sherman PM, Holko M, Yefanov A, Lee H, Zhang N, Robertson CL, Serova N, Davis S, Soboleva A (2013). NCBI GEO: archive for functional genomics data sets—update. Nucleic Acids Res.

[28] Tarazona S, García-Alcalde F, Dopazo J, Ferrer A, Conesa A (2011). Differential expression in RNA-seq: a matter of depth. Genome Res.

[29] Djebali S, Davis CA, Merkel A, Dobin A, Lassmann T, Mortazavi A, Tanzer A, Lagarde J, Lin W, Schlesinger F, Xue C, Marinov GK, Khatun J, Williams BA, Zaleski C, Rozowsky J, Röder M, Kokocinski F, Abdelhamid RF, Alioto T, Antoshechkin I, Baer MT, Bar NS, Batut P, Bell K, Bell I, Chakrabortty S, Chen X, Chrast J, Curado J (2012). Landscape of transcription in human cells. Nature.

[30] Erhard F, Lieber D, Malterer G, Jaskiewicz L, Zavolan M, Do L, Zimmer R (2014). Widespread context dependency of microRNA- mediated regulation. Genome Res.

[31] R: A Language and Environment for Statistical Computing [http://www.r-project.org/]

[32] Irizarry RA, Hobbs B, Collin F, Beazer-Barclay YD, Antonellis KJ, Scherf U, Speed TP (2003). Exploration, normalization, and summaries of high density oligonucleotide array probe level data. Biostatistics.

[33] Gautier L, Cope L, Bolstad BM, Irizarry RA (2004). affy--analysis of Affymetrix GeneChip data at the probe level. Bioinformatics.

[34] Smyth GK (2004). Linear models and empirical Bayes methods for assessing differential expression in microarray experiments. Stat Appl Genet Mol Biol.

[35] Cortes C, Vapnik V (1995). Support-Vector Networks. Mach Learn.

[36] Chang C-C, Lin C-J (2011). LIBSVM : A Library for Support Vector Machines. ACM Trans Intell Syst Technol.

[37] Metz CE (1978). Basic Principles of ROC Analysis. Semin Nucl Med.

[38] Hanley JA, McNeil BJ (1982). The meaning and use of the area under a receiver operating characteristic (ROC) curve. Radiology.

[39] Robin X, Turck N, Hainard A, Tiberti N, Lisacek F, Sanchez J-C, Müller M (2011). pROC: an open-source package for R and S+ to analyze and compare ROC curves. BMC Bioinformatics.

[40] Fawcett T (2006). An introduction to ROC analysis. Pattern Recognit Lett.

[41] Using the SRA Toolkit to convert.sra files into other formats [http://www.ncbi.nlm.nih.gov/books/NBK158900/]

[42] Davis MP, van Dongen S, Abreu-Goodger C, Bartonicek N, Enright AJ (2013). Kraken: a set of tools for quality control and analysis of high-throughput sequence data. Methods.

[43] Flicek P, Amode MR, Barrell D, Beal K, Billis K, Brent S, Carvalho-Silva D, Clapham P, Coates G, Fitzgerald S, Gil L, Girón CG, Gordon L, Hourlier T, Hunt S, Johnson N, Juettemann T, Kähäri AK, Keenan S, Kulesha E, Martin FJ, Maurel T, McLaren WM, Murphy DN, Nag R, Overduin B, Pignatelli M, Pritchard B, Pritchard E, Riat HS (2014). Ensembl 2014. Nucleic Acids Res.

[44] Langmead B, Salzberg SL (2012). Fast gapped-read alignment with Bowtie 2. Nat Methods.

[45] Lawrence M, Huber W, Pagès H, Aboyoun P, Carlson M, Gentleman R, Morgan MT, Carey VJ (2013). Software for Computing and Annotating Genomic Ranges. PLoS Comput Biol.

[46] Robinson MD, McCarthy DJ, Smyth GK (2010). edgeR: a Bioconductor package for differential expression analysis of digital gene expression data. Bioinformatics.

[47] Vigorito E, Perks KL, Abreu-Goodger C, Bunting S, Xiang Z, Kohlhaas S, Das PP, Miska EA, Rodriguez A, Bradley A, Smith KG, Rada C, Enright AJ, Toellner KM, Maclennan IC, Turner M (2007). microRNA-155 regulates the generation of immunoglobulin class-switched plasma cells. Immunity.

[48] Lu D, Nakagawa R, Lazzaro S, Staudacher P, Abreu-goodger C, Henley T, Boiani S, Leyland R, Galloway A, Andrews S, Butcher G, Nutt SL, Turner M, Vigorito E (2014). The miR-155 – PU.1 axis acts on Pax5 to enable efficient terminal B cell differentiation. J Exp Med.

[49] Bray NL, Pimentel H, Melsted P, Pachter L: Near-optimal RNA-Seq quantification. aRxiv 2015.

[50] Law CW, Chen Y, Shi W, Smyth GK (2014). voom: Precision weights unlock linear model analysis tools for RNA-seq read counts. Genome Biol.

[51] Farrell CM, O’Leary NA, Harte RA, Loveland JE, Wilming LG, Wallin C, Diekhans M, Barrell D, Searle SMJ, Aken B, Hiatt SM, Frankish A, Suner MM, Rajput B, Steward CA, Brown GR, Bennett R, Murphy M, Wu W, Kay MP, Hart J, Rajan J, Weber J, Snow C, Riddick LD, Hunt T, Webb D, Thomas M, Tamez P, Rangwala SH (2014). Current status and new features of the Consensus Coding Sequence database. Nucleic Acids Res.

[52] Michaud J, Simpson KM, Escher R, Buchet-Poyau K, Beissbarth T, Carmichael C, Ritchie ME, Schütz F, Cannon P, Liu M, Shen X, Ito Y, Raskind WH, Horwitz MS, Osato M, Turner DR, Speed TP, Kavallaris M, Smyth GK, Scott HS (2008). Integrative analysis of RUNX1 downstream pathways and target genes. BMC Genomics.

[53] Huang DW, Sherman BT, Lempicki RA (2009). Systematic and integrative analysis of large gene lists using DAVID bioinformatics resources. Nat Protoc.

[54] Huang DW, Sherman BT, Lempicki RA (2009). Bioinformatics enrichment tools: Paths toward the comprehensive functional analysis of large gene lists. Nucleic Acids Res.

[55] Liu W, Mei R, Di X, Ryder TB, Hubbell E, Dee S, Webster TA, Harrington CA, Ho M, Baid J, Smeekens SP (2002). Analysis of high density expression microarrays with signed-rank call algorithms. Bioinformatics.

[56] van Rooij E, Sutherland LB, Thatcher JE, DiMaio JM, Naseem RH, Marshall WS, Hill JA, Olson EN. Dysregulation of microRNAs after myocardial infarction reveals a role of miR-29 in cardiac fibrosis. Proc Natl Acad Sci U S A. 2008;105:13027–32. 10.1073/pnas.0805038105 PMC2529064 18723672

[57] Small EM, Olson EN (2011). Pervasive roles of microRNAs in cardiovascular biology. Nature.

[58] Kole AJ, Swahari V, Hammond SM, Deshmukh M (2011). miR-29b is activated during neuronal maturation and targets BH3-only genes to restrict apoptosis. Genes Dev.

[59] Shi G, Liu Y, Liu T, Yan W, Liu X, Wang Y, Shi J, Jia L (2012). Upregulated miR-29b promotes neuronal cell death by inhibiting Bcl2L2 after ischemic brain injury. Exp Brain Res.

