# Supplementary material for: Improving microRNA target prediction with gene expression profiles
Source: BMC Genomics. 2016 May 17;17:364. doi: 10.1186/s12864-016-2695-1 (PMC4869178; doi:10.1186/s12864-016-2695-1)
Supplement: Supplementary file 1 — Supplementary Material. This file contains Tables S1–S3 and Figures S1-S11. These include full descriptions and QC of the microarray and RNA-Seq experiments that we analysed, the number of training genes contributing to the TargetExpress model, expression bias of functional miRNA targets, Sylamer seed-enrichment results, additional performance comparisons between target prediction models (AUC, sensitivity, specificity and ROC curves) and enriched GO Terms suggested by miR-29 literature. (PDF 1937 kb) [file 12864_2016_2695_MOESM1_ESM.pdf]

Supplementary Table 1

Experimental datasets analyzed to train *n* SVM classification models (TargetExpress).

Experiment (GEO ID)	Treatment samples name	Control samples name	Contrast	Sylamer max(-log10(Syl-P value))	Sylamer good signal quality	Transcripts with miRNA seed FDR <= 0.1 and t < 0 (True Targets)	Transcripts with miRNA seed FDR >= 0.2 (False Targets)	Used as one of the *n* SVM models for TargetExpress prediction?
**Human microarray experiments**								
GSE19737 – AACTGGAA, miR145	miR_145_overexpressing_group	Control_group	miR_145_overexpressing_group-Control_group	26.54	Yes	79	6475	Yes
GSE13460 – ACACTCCA, miR122	HES2_Mir122WT	HES2_Mir122Mut	HES2_Mir122WT - HES2_Mir122Mut	0.58	No	0	6542	No
GSE45627 – ATGTAGCA, miR-221	pre221	ctrl	pre221 - ctrl	21.27	Yes	0	6544	No
GSE7754 – CACTGCCA, miR-34a	HCT116_cells_with_enforced_miR_34a_expression	HCT116_cells_with_empty_vector	HCT116_cells_with_enforced_miR_34a_expression - HCT116_cells_with_empty_vector	2.05	No	2221	4732	No
GSE33538 – GCACTTTA, miR-20a (miR-17/20a)	si_control_at	si_miR_20a_a	si_control_at - si_miR_20a_a	9.77	Yes	254	9705	Yes
GSE33538 – GGCTTCCA, miR-671-5p	si_control_at	si_miR_671_at	si_control_at - si_miR_671_at	0.64	No	217	10588	No
GSE14507 – GTCTTCCA, miR-7	A549_miR_7	A549_miR_NC	A549_miR_7-A549_miR_NC	60.18	Yes	80	8414	Yes
GSE18651 – TGGTGCTA, miR-29	IMR_90_SCR_100nM_KD	IMR_90_miR_29_KD_100nM, IMR_90_miR_29_KD_50nM	IMR_90_SCR_100nM_KD-(IMR_90_miR_29_KD_100nM+IMR_90_miR_29_KD_50nM)/2	22.11	Yes	857	4399	Yes
GSE27718 – TGTTTACA, miR-30a	4L_melanoma_cell_line_miR_30d_treated_sample_repeat, 5B1_melanoma_cell_line_miR_30d_treated_sample_repeat	4L_melanoma_cell_line_scrambled_treated_sample_repeat, 5B1_melanoma_cell_line_scrambled_treated_sample_repeat	"(4L_melanoma_cell_line_miR_30d_treated_sample_repeat+5B1_melanoma_cell_line_miR_30d_treated_sample_repeat)/2 - (4L_melanoma_cell_line_scrambled_treated_sample_repeat+5B1_melanoma_cell_line_scrambled_treated_sample_repeat)/2	47.20	Yes	32	7832	Yes
**Mouse microarray experiments**								
GSE13590 – AACCACTA, miR-140-5p	siRNA_140	antimicroRNA_140	siRNA_140 - antimicroRNA_140	7.26	Yes	913	7645	Yes
GSE13948 – ACACTCCA, miR-122	p0617_control_day_3, p0617_control_day_9	p0617_treated_day_3, p0617_treated_day_	"(p0617_control_day_3+p0617_control_day_9)/2-(p0617_treated_day_3+p0617_treated_day_9)/2	24.25	Yes	855	7403	Yes
GSE18840 – AGCACTTA, miR-294 (miR-302)	miR_294_transfected	mock_transfected	miR_294_transfected-mock_transfected	39.62	Yes	2235	4754	Yes
GSE18840 – CTACCTCA, let-7	let_7c_transfected	mock_transfected	let_7c_transfected-mock_transfected	52.07	Yes	1349	5471	Yes
E-MTAB-2038 – GGCAGCTA (miR-22)	Heart_WT	Heart_KO	Heart_WT-Heart_KO	10.94	Yes	11	4933	No

E-MTAB-2038 – GGCAGCTA (miR-22)	Kidney_WT	Kidney_KO	Kidney_WT-Kidney_KO	8.72	Yes	26	2242	Yes
E-MTAB-2038 – GGCAGCTA (miR-22)	Liver_WT	Liver_KO	Liver_WT-Liver_KO	6.65	Yes	62	4767	Yes
E-MTAB-2038 – GGCAGCTA (miR-22)	Lung_WT	Lung_KO	Lung_WT-Lung_KO	2.54	No	77	1698	No
E-MTAB-2038 – GGCAGCTA (miR-22)	Muscle_WT	Muscle_KO	Muscle_WT-Muscle_KO	8.67	Yes	21	5155	Yes
E-MTAB-2038 – GGCAGCTA (miR-22)	Wat_WT	Wat_KO	Wat_WT-Wat_KO	3.67	No	30	2115	No
GSE57511 – TGAATGTA, miR-181a	Dicer_lox_lox_control	LckCre_Dicer_lox_lox_KO	Dicer_lox_lox_control - LckCre_Dicer_lox_lox_KO	3.84	No	3	9988	No
GSE27035– TGGTGCTA, miR-29a	Astrocyte_miR_29_transfected	Astrocyte_control_transfected	Astrocyte_miR_29_transfected - Astrocyte_control_transfected	29	Yes	2638	1964	Yes
E-MEXP-1325 – TAGCATTA, miR-155	WT	KO	WT - KO	16	Yes	101	4502	NO
**Mouse RNA-seq experiments**								
SRP047132/ GSE61425 – TAGCATTA, miR-155	WT	KO	WT - KO	24	Yes	1143	840	NO
**Zebrafish microarray experiments**								
GSE12991 – ACATTCCA, miR-1	wt_206_GFP_pos	wt_206_GFP_neg	wt_206_GFP_pos - wt_206_GFP_neg	9.50	Yes	2660	1946	No
GSE12991 – GTGCCTTA, miR-124	wt_124_GFP_pos	wt_124_GFP_neg	wt_124_GFP_pos - wt_124_GFP_neg	10.38	Yes	3978	1309	No
GSE4201 – AGCACTTA, miR-430 (miR-302)	Zebrafish_MZ_Dicer_miR_430_injected	Zebrafish_MZ_Dicer	Zebrafish_MZ_Dicer_miR_430_injected - Zebrafish_MZ_Dicer	27.16	Yes	3763	1399	No
**Zebrafish RNA-seq experiments**								
SRP010040/ GSE34743	mRNA-seq-WT	mRNA-seq-MZDicer	mRNA-seq-WT - mRNA-seq-MZDicer	12	Yes	494	4189	No
SRP010040/ GSE34743	Ribosome-profilling-WT	Ribosome-profilling-MZDicer	Ribosome-profilling-WT - Ribosome-profilling-MZDicer	13	Yes	164	2670	No

Supplementary Table 2

Independent expression samples used to train each SVM classification model given experimental condition.

Experiment (GEO ID)	Expression samples	Independent samples from experiment?
GSE19737 – AACTGGAA, miR145	GSM320837 and GSM320842 from GSE12791	Yes
GSE33538 – GCACTTTA, miR-20a (miR-17)	GSM469159 and GSM469160 from GSE18938	Yes
GSE14507 – GTCTTCCA, miR-7	GSM803648 and GSM803707 from GSE32474	Yes
GSE18651 – TGGTGCTA, miR-29	GSM463331 and GSM463332 from GSE18651	No
GSE27718 – TGTTTACA, miR-30a	GSM686341 and GSM686345 from GSE27718	No
GSE13590 – AACCACTA, miR-140-5p	GSM341737 and GSM341739 from GSE13590	No
GSE13948 – ACACTCCA, miR-122	GSM967009 and GSM967011 from GSE39375	Yes
GSE18840 – AGCACTTA, miR-294 (miR-302)	GSM638129 and GSM638130 from GSE26001	Yes
GSE18840 – CTACCTCA, let-7	GSM638131 and GSM638132 from GSE26001	Yes
E-MTAB-2038 – GGCAGCTA (miR-22) kidney	GSM252083 and GSM252084 from GSE9954	No
E-MTAB-2038 – GGCAGCTA (miR-22) liver	GSM252074 and GSM252075 from GSE9954	No

Supplementary Table 3

Number of training genes from all perturbation experiments used to train TargetExpress contributing to predict True Targets.

Score	[-1,-0.5]	[-0.5,0]	[0,0.5]	[0.5,1]	NoInfo
Expression	792	2423	9631	16112	0
Total_context_score	1906	2008	2873	4448	17714
miTG	1931	2128	3048	5363	16478
PCT	1033	1026	1471	2066	23350
MIRZA	1847	2395	2927	3651	18134
MIRZA-F	2441	2211	2611	3555	18134
MIRZA-N	2647	3155	3926	4712	14520

Supplementary Figure 1

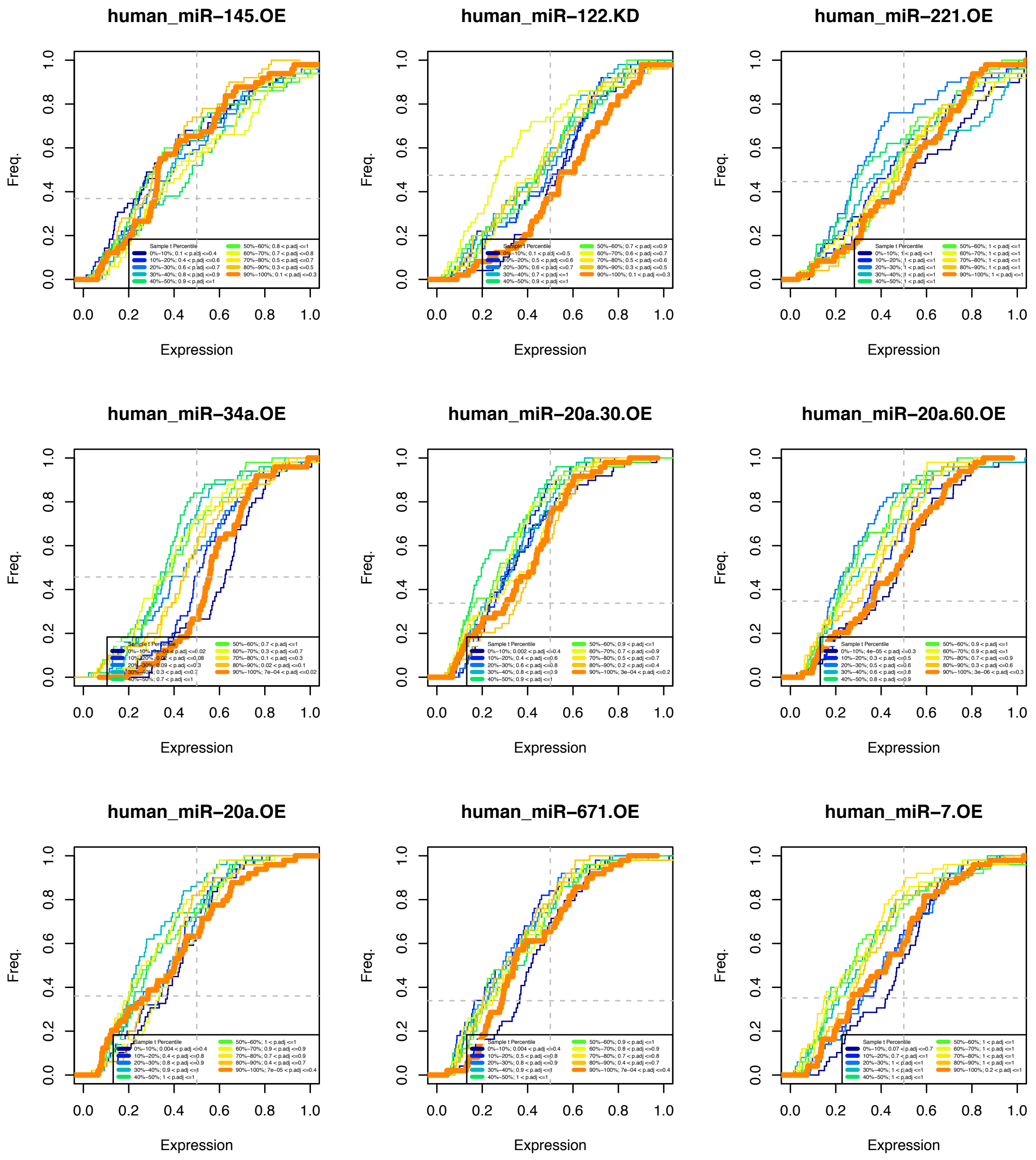

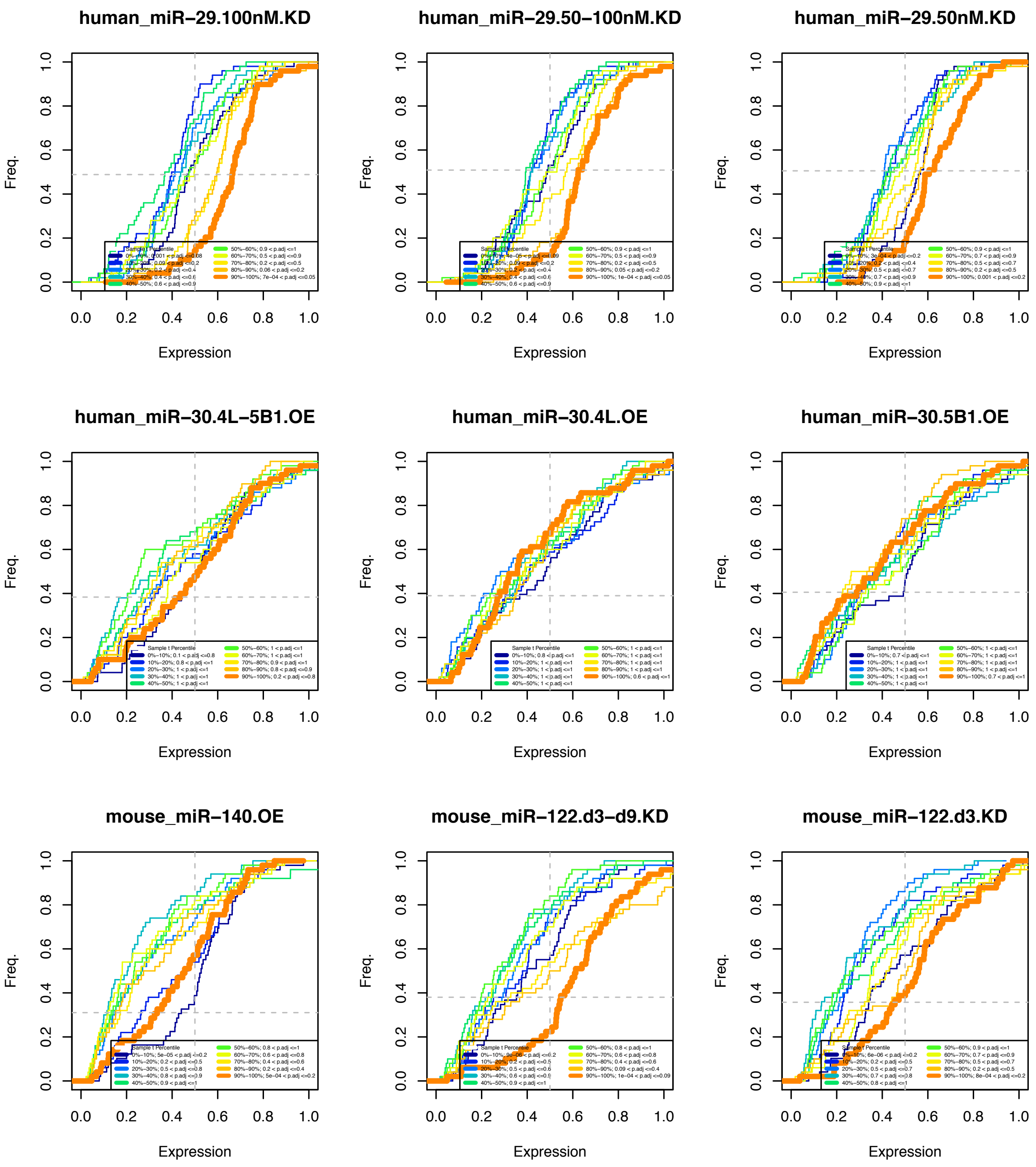
human_miR−29.100nM.KD
human_miR−29.50−100nM.KD
human_miR−29.50nM.KD
human_miR−30.4L−5B1.OE
human_miR−30.4L.OE
human_miR−30.5B1.OE
mouse_miR−140.OE
mouse_miR−122.d3−d9.KD
mouse_miR−122.d3.KD
Freq.
Expression
0.0
0.2
0.4
0.6
0.8
1.0
Sample t Percentile

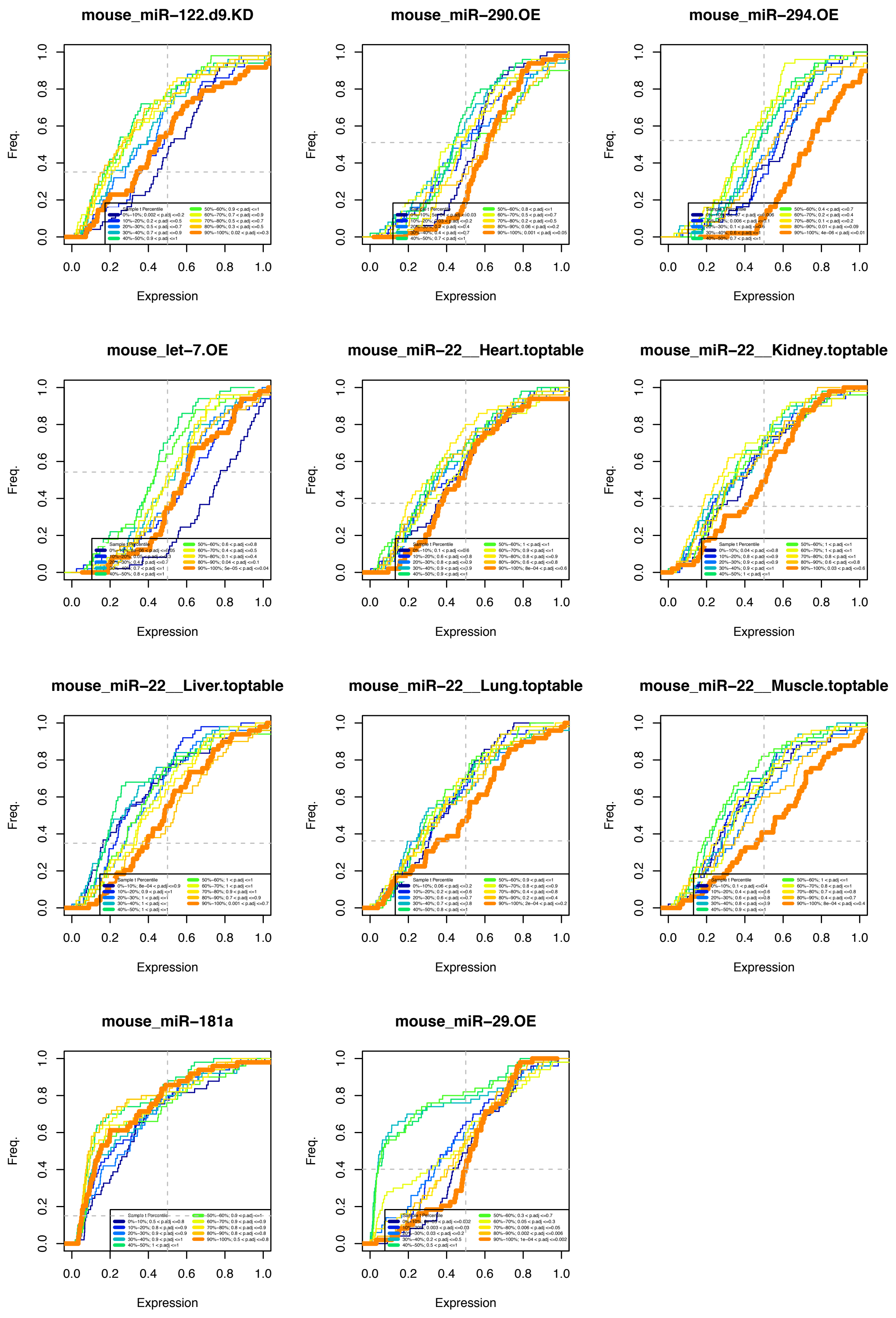

Expression bias of functional microRNA targets. For several microRNA perturbation experiments, we show the empirical cumulative distributions of scaled expression values. All genes are split according to their differential expression into 10 groups. More orange colors represent genes that are being repressed in presence of the miRNA, while more blue colors represent genes that are being activated in presence of the miRNA. Green colors are those that are not significantly changing. In general, the most affected genes tend to have higher expression than genes that do not change.

Supplementary Figure 2

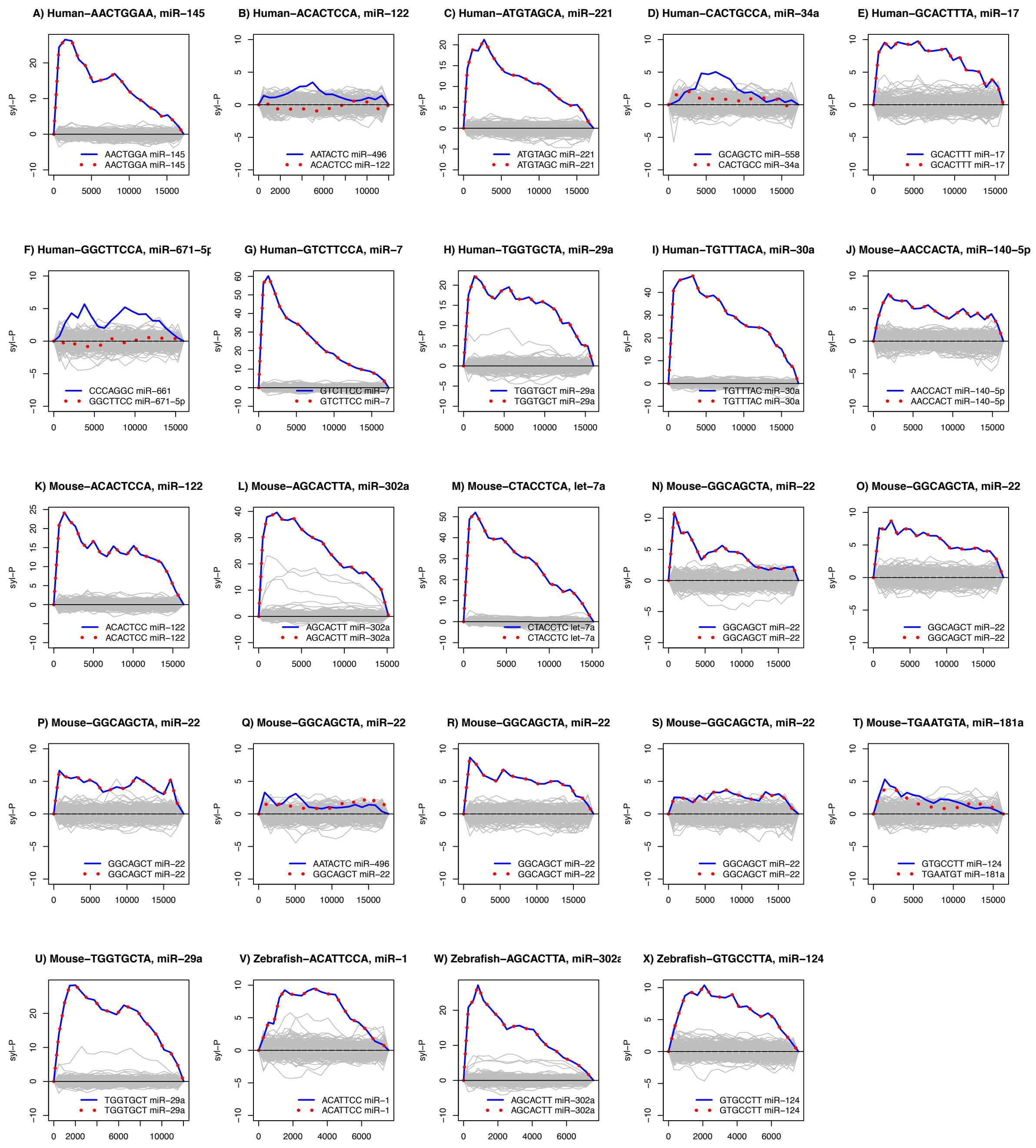

Sylamer plots. The 3'UTR sequences are sorted according to differential expression in each experiment (see Methods) and the Sylamer algorithm is used to find enriched 7mer motifs. The blue line denotes the most significant 7mer and red dotted line indicates the 7mer matching the seed of the perturbed miRNA.

Supplementary Figure 3

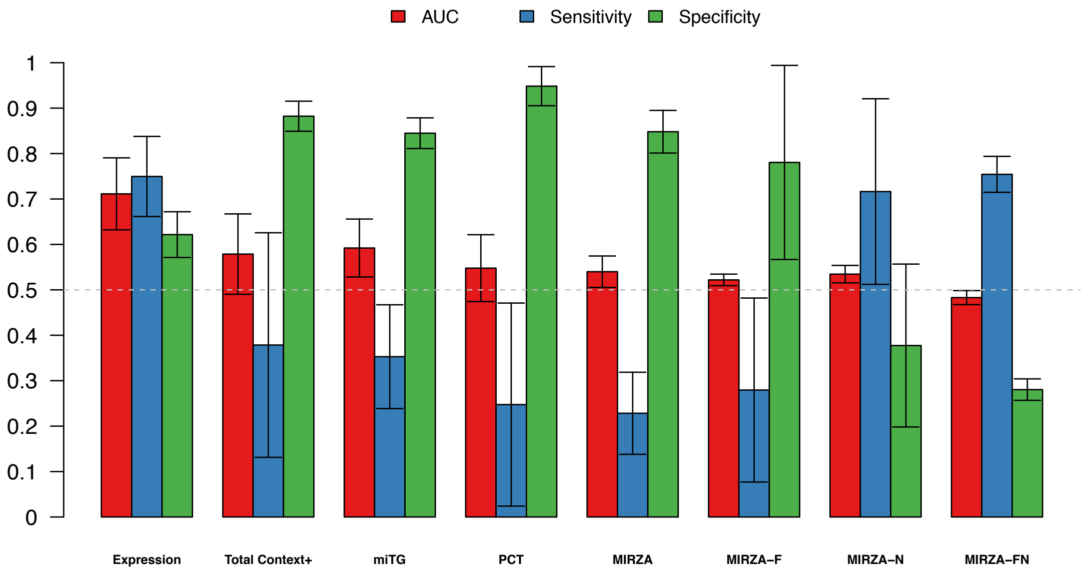

Feature model contribution classification values. Area Under the Curve (AUC, red), sensitivity (blue) and specificity (green) mean values and its standard deviation for each feature: microArray Expression, microT-CDS's miTG, Target Scan's Total context score, Target Scan's Probability of Conserved Targeting (PCT), MIRZA canonical sites (MIRZA), MIRZA target frequency (MIRZA-F), MIRZA non canonical sites (MIRZA-N) and MIRZA target frequency non canonical sites (MIRZA-FN). Higher AUCs indicate more contribution to the SVM classification.

Supplementary Figure 4

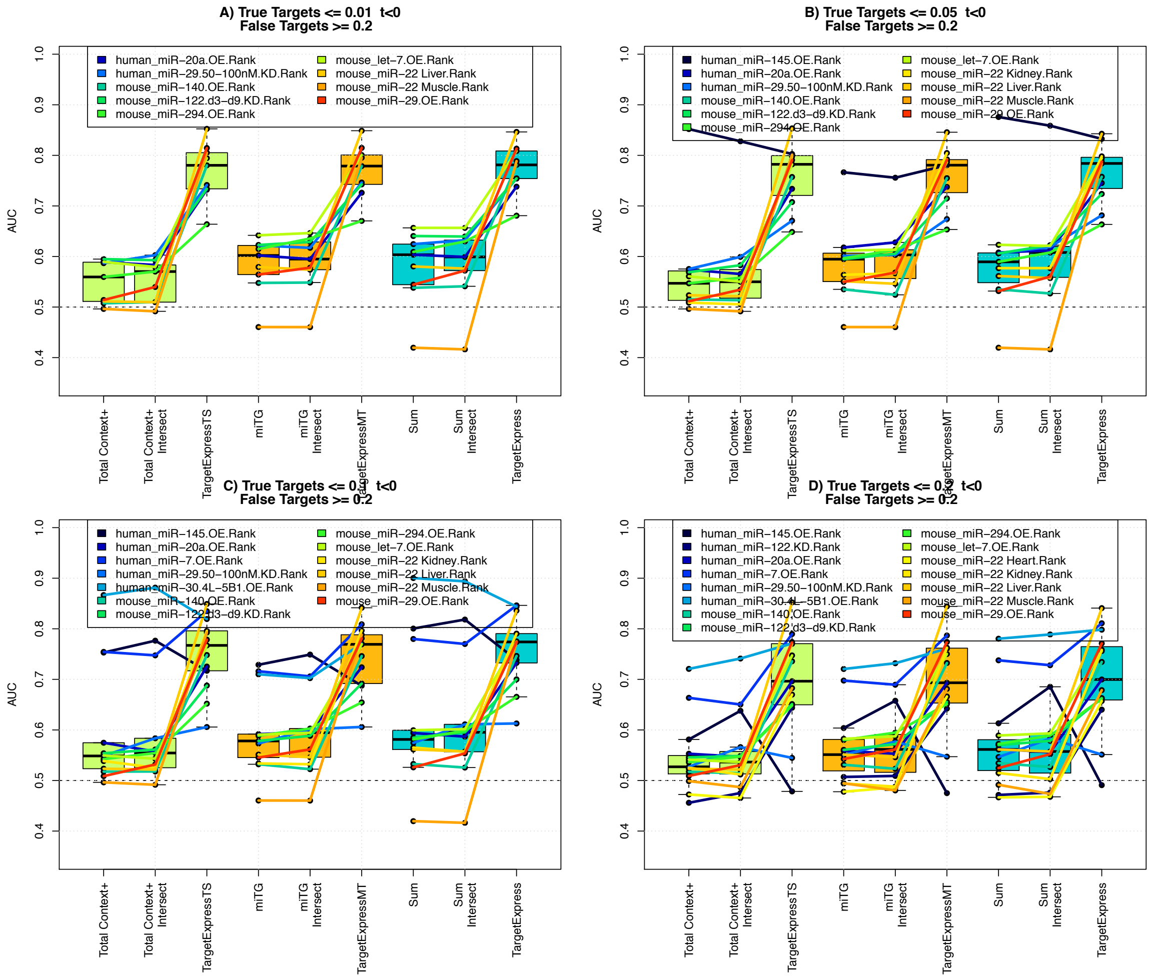

Leave one out experiment cross-validation. The area under the curve (AUC) for each “left out” experiment (indicated in the top legend) given different prediction models: TargetScan, TargetScan-intersect and TargetExpress-TS (green boxplots); microT, microT-intersect and TargetExpress-MT (orange); Sum, Sum-intersect and TargetExpress-Sum (blue). (A-D) Different FDR cutoffs are used to define the True Targets (see Methods).

Supplementary Figure 5

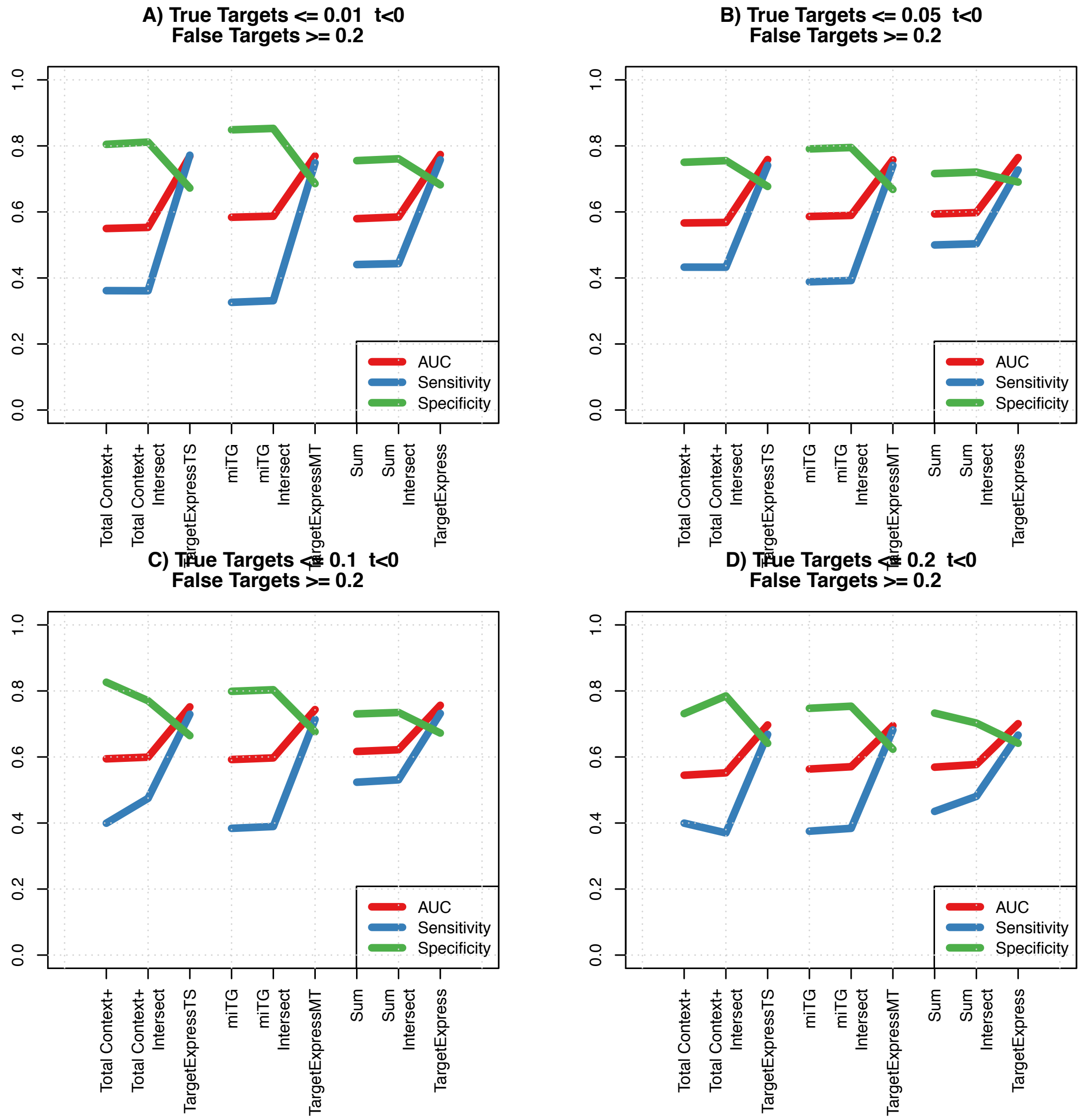

Mean classification values for leave one out experiment cross-validation. Mean values of Area Under the Curve (AUC), sensitivity and specificity (AUC, red line, sensitivity, blue line, and specificity, green line, see Methods) were calculatedfor different FDR cutoffs used to define the True Targets (see Methods).

Supplementary Figure 6

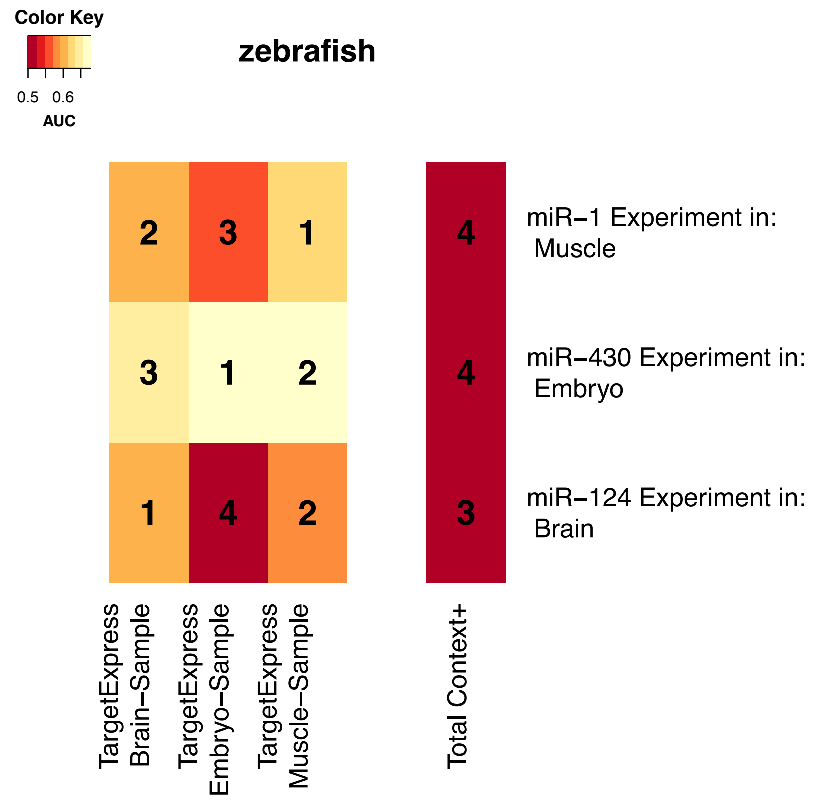

Tissue specificity of predictions in zebrafish experiments. Three predictions (three different tissues) for each miRNA (dre-miR-1, dre-miR124, dre-miR4-30) are obtained, given TargetExpress, and the respective Target Scan prediction. Each prediction is evaluated using AUC metric given True/False targets for zebrafish experiment (GSE12991, GSE4201).

Supplementary Figure 7

A) WT6H_vs_MZ6H

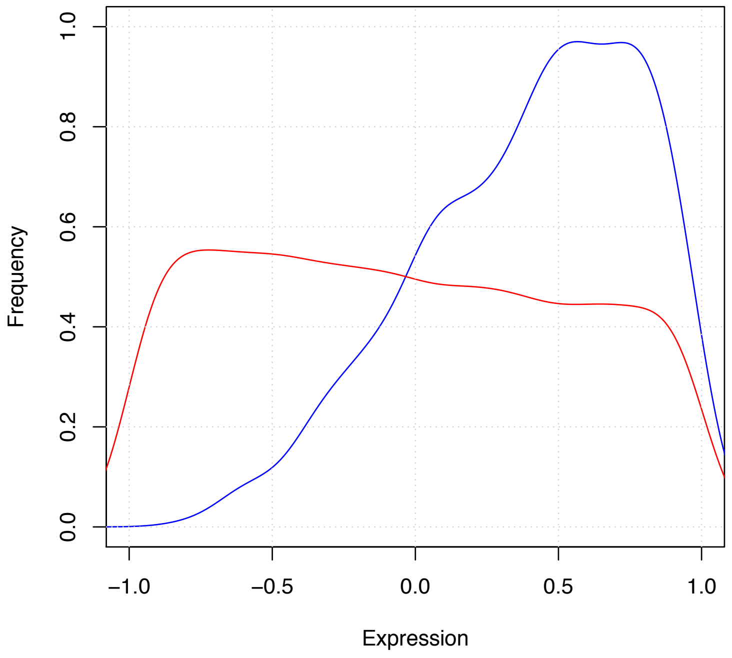

B) RiboWT6H_vs_RiboMZ6H

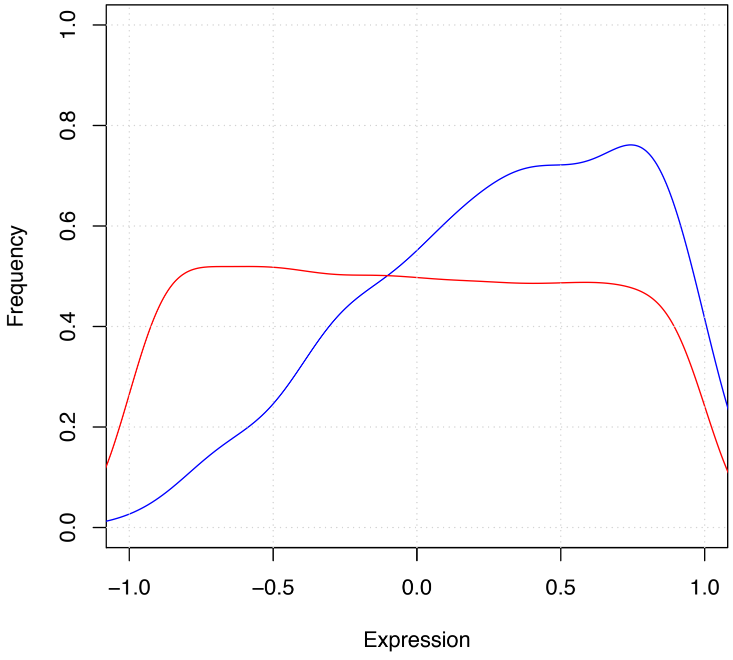

Expression bias on MZDicer high-throughput experiments. Most True targets (blue) are highly expressed compared to False targets (red), measured according to A) RNA-Seq or B) Ribosome-profiling.

Supplementary Figure 8

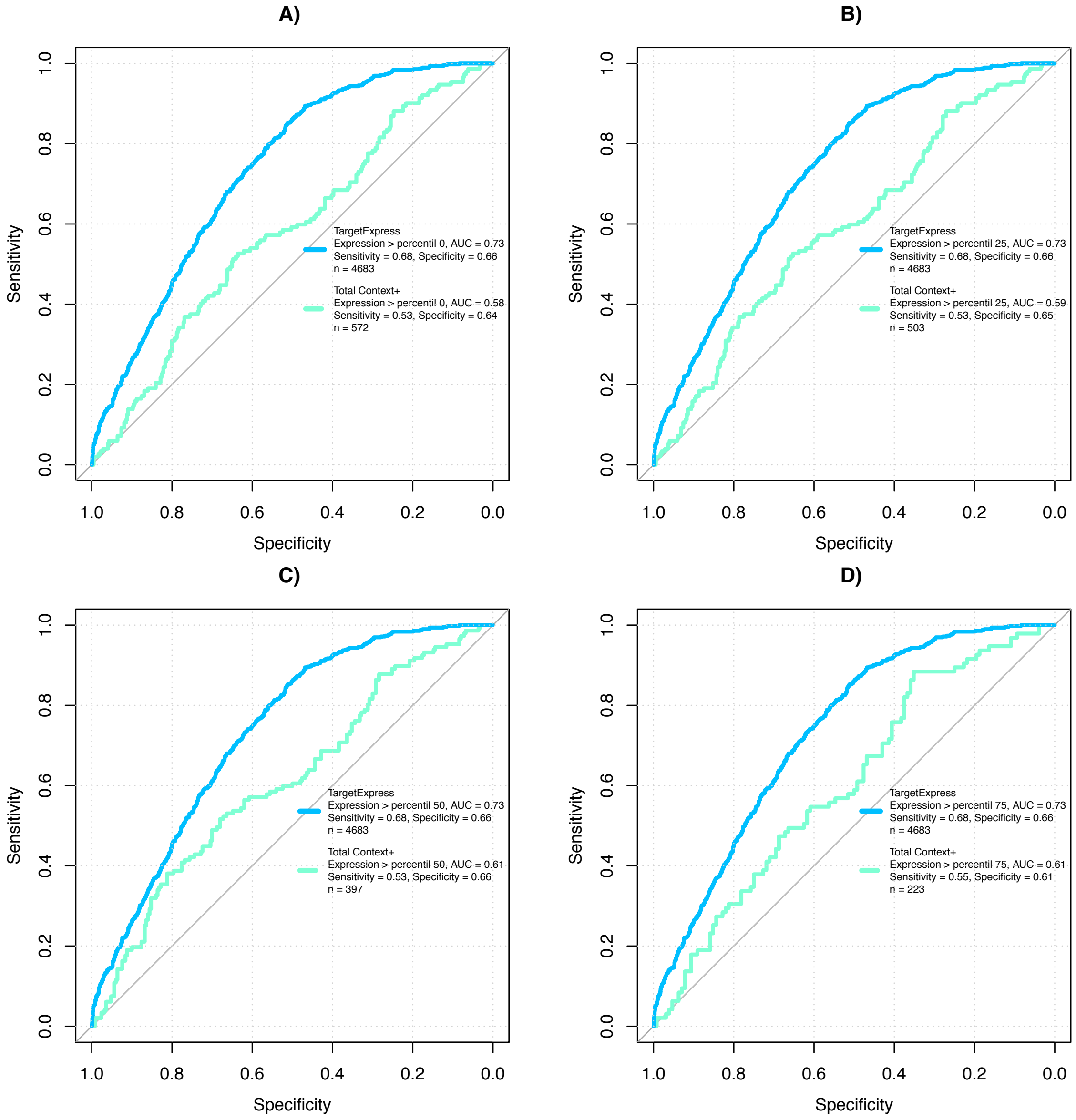

ROC curves for miR-430 targets in zebrafish embryos using mRNA profiling. Performance comparison using mRNA expression profiling data of zebrafish embryos. We have two different target prediction approaches (TargetScan scores for targets with expression higher than expression percentiles 0 (A), 25 (B), 50 (C) and 75 (D) and TargetExpress scores A-D). We compared them with the Area Under the Receiving Operator Curve (ROC).

Supplementary Figure 9

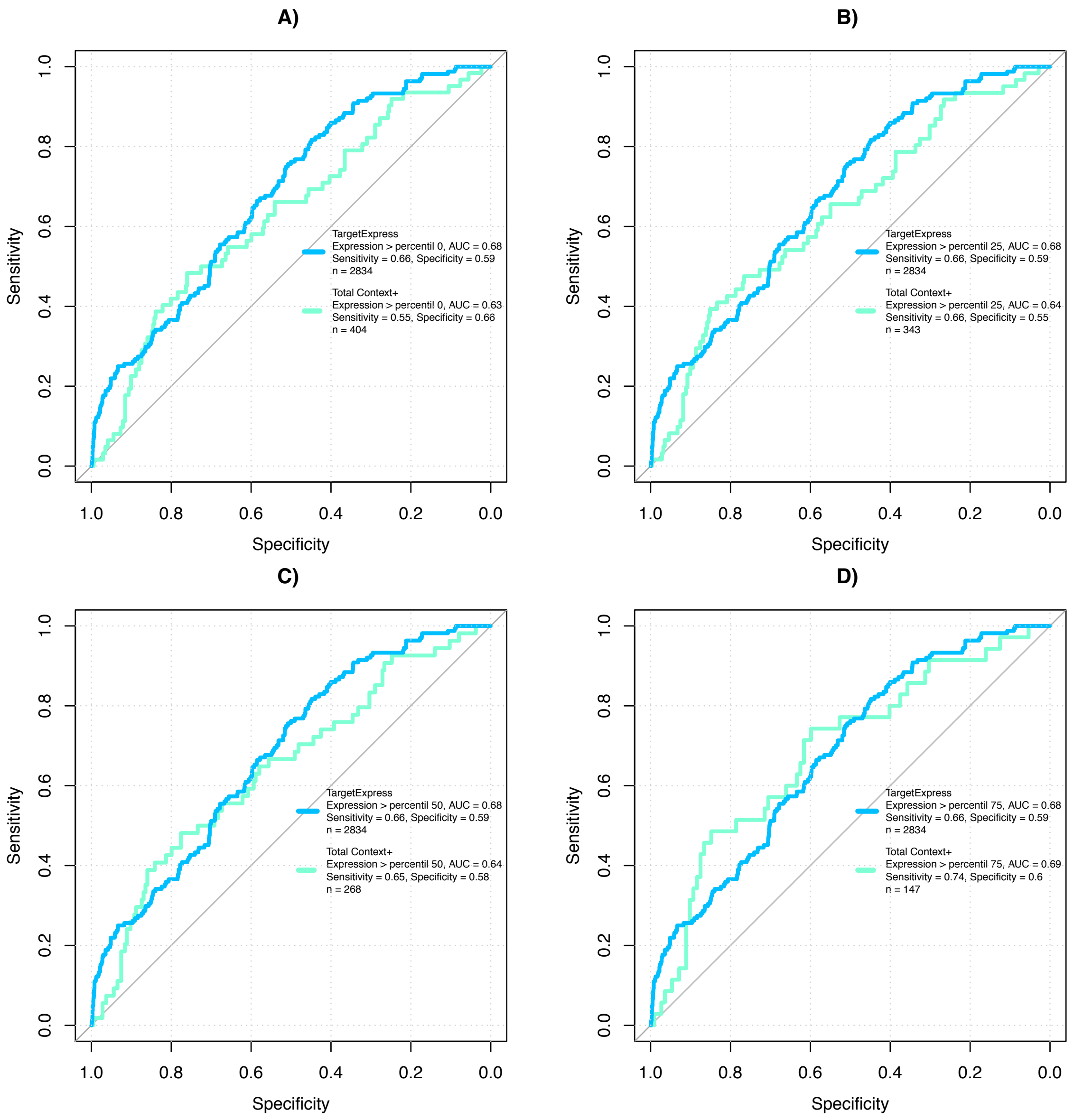

ROC curves for miR-430 targets in zebrafish embryos using ribosome profiling. Performance comparison using ribosome expression profiling data of zebrafish embryos. We have two different target prediction approaches (TargetScan scores for targets with expression higher than expression percentiles 0 (A), 25 (B), 50 (C) and 75 (D) and TargetExpress scores A-D). We compared them with the Area Under the Receiving Operator Curve (ROC).

Supplementary Figure 10

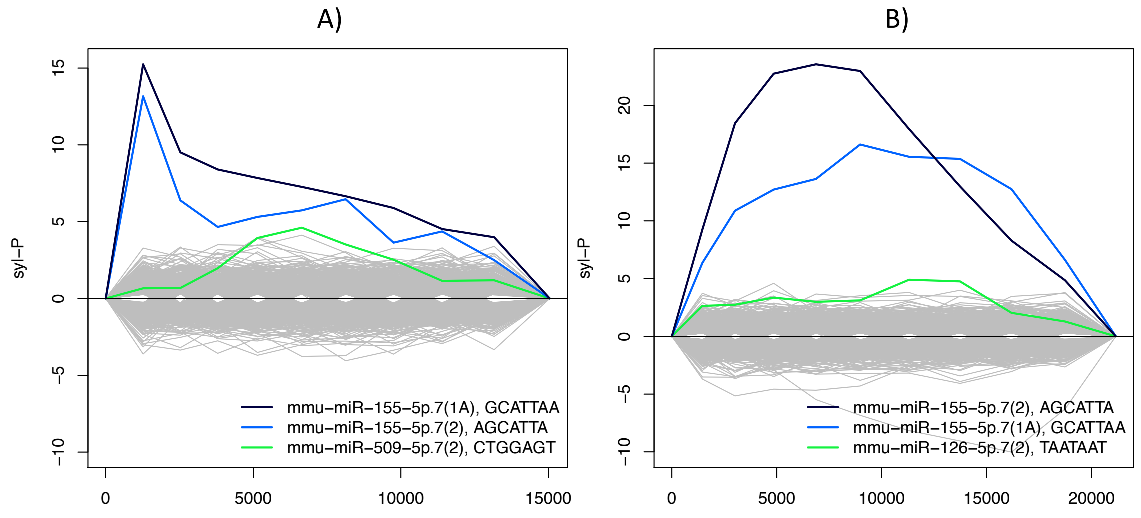

Sylamer plots comparing TargetExpress predictions using microarray and RNA-seq expression profiles. The 3'UTR sequences are sorted according to differential expression (mmu-miR-155-5p perturbation) in each experiment, A) microarray and B) RNA-Seq (see Methods) and the Sylamer algorithm is used to find enriched 7mer motifs. Black and blue lines denote the most significant 7mers. Both lines match the seed of the perturbed miRNA.

Supplementary Figure 11

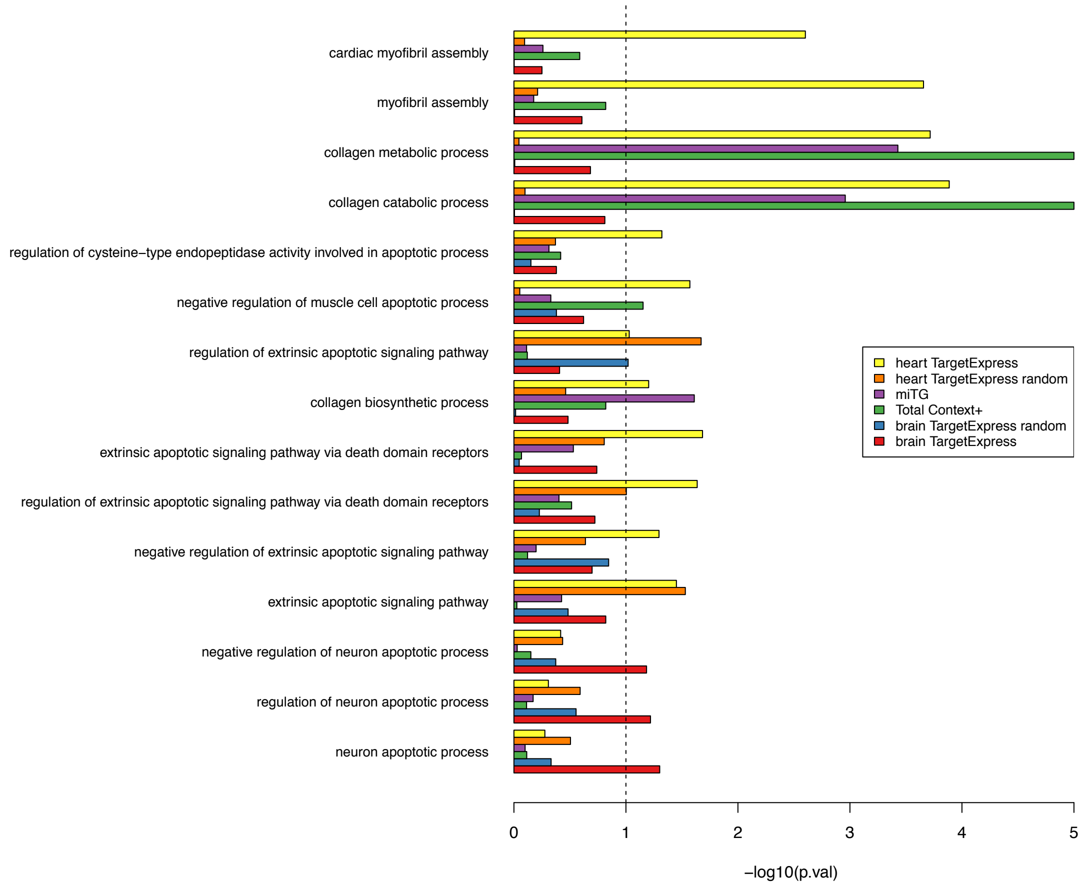

Enrichment of GO terms suggested by literature. GO term enrichment comparing predictions for miR-29 in heart and brain tissues. GO enrichment for TargetExpress predictions are shown in yellow (heart) and red (brain) bars, for random TargetExpress predictions are shown in orange (heart random) and blue (brain random). GO enrichment values for microT-CDS and TargetScan predictions are shown in purple and green bars, respectively. GO terms shown were selected searching key words “fibrosis”, “collagen”, “apoptosis”.
